# Comparative analysis of mesenchymal stem cells cultivated in serum free media

**DOI:** 10.1038/s41598-022-12467-z

**Published:** 2022-05-21

**Authors:** Joo Youn Lee, Min Hee Kang, Ji Eun Jang, Jeong Eon Lee, Yuyeong Yang, Ji Yong Choi, Hong Seok Kang, Uiil Lee, Ji Woong Choung, Hyeryeon Jung, Young-Chan Yoon, Kyung Hee Jung, Soon–Sun Hong, Eugene C. Yi, Sang Gyu Park

**Affiliations:** 1 Xcell Therapeutics, Dongwon Bldg. 6F, 333, Yeongdong-daero, Gangnam-gu, Seoul, 06188 Korea; 2 Dacapo Oral & Maxillofacial Surgery Clinic, Jeongin Building, 559 Gangnamdae-ro, Seocho-gu, Seoul, 06531 Korea; 3 Department of Molecular Medicine and Biopharmaceutical Sciences, Graduate School of Convergence Science and Technology and College of Medicine Or College of Pharmacy, Seoul National University, Seoul, 03080 Korea; 4 Department of Medicine, College of Medicine, Inha University, 27 Inhang-ro, Jung-gu, Incheon, 22332 Korea; 5 Department of Pharmacy, College of Pharmacy, Ajou University, Worldcup-ro, 206, Yeongtong-gu, Suwon, 16499 Korea

**Keywords:** Cell biology, Stem cells

## Abstract

Stem cells are attractive candidates for the regeneration of tissue and organ. Mesenchymal stem cells (MSCs) have been extensively investigated for their potential applications in regenerative medicine and cell therapy. For developing effective stem cell therapy, the mass production of consistent quality cells is required. The cell culture medium is the most critical aspect of the mass production of qualified stem cells. Classically, fetal bovine serum (FBS) has been used as a culture supplement for MSCs. Due to the undefined and heterologous composition of animal origin components in FBS, efforts to replace animal-derived components with non-animal-derived substances led to safe serum free media (SFM). Adipose derived mesenchymal stem cells (ADSCs) cultivated in SFM provided a more stable population doubling time (PDT) to later passage and more cells in a shorter time compared to FBS containing media. ADSCs cultivated in SFM had lower cellular senescence, lower immunogenicity, and higher genetic stability than ADSCs cultivated in FBS containing media. Differential expression analysis of mRNAs and proteins showed that the expression of genes related with apoptosis, immune response, and inflammatory response were significantly up-regulated in ADSCs cultivated in FBS containing media. ADSCs cultivated in SFM showed similar therapeutic efficacy in an acute pancreatitis mouse model to ADSCs cultivated in FBS containing media. Consideration of clinical trials, not only pre-clinical trial, suggests that cultivation of MSCs using SFM might offer more safe cell therapeutics as well as repeated administration due to low immunogenicity.

## Introduction

Mesenchymal stem cells (MSCs) are attractive for allogeneic cell based therapies because of their ability to secrete growth factors, differentiate into many types of cells, their anti-inflammatory role^1–7^. Human adipose-derived mesenchymal stem cells (ADSCs) are representative MSC sources for cell therapy. However, a standard expansion method for ADSCs of consistent quality is necessary before clinical studies. The culture medium is an important aspect of the mass production of qualified cells. Despite the limitations of fetal bovine serum (FBS) to in vitro manipulation of ADSCs for clinical trial and its potential risks of unidentified viral infection, immunological reaction and batch/lot variation^8–10^, this xenogenic additive is still allowed. A recent study detected cell contamination due to the internalization of fetal calf serum (FCS) origin antigens into MSCs cultured in an FCS containing medium, even after the washing step^11^. Bovine apolipoprotein B-100 was the dominant immunogen in therapeutic cells cultured with FCS^12^. Other reports showed that hMSC and human embryonic stem cells cultured with FBS could express N-glycolylneuraminic acid (Neu5Gc) xenoantigen, even though humans cannot synthesize Neu5Gc. Cellular products that express Neu5Gc could have compromised viability and efficacy because human serum contains antibodies anainst Neu5Gc^13,14^. Besides the potential to elicit immune reactions in human patients, animal derived reagents pose potential risks of transmitting zoonotic viral or prion diseases^14–17^.

To address these concerns, human supplements, including human serum and platelet lysate (HPL), have been proposed to replace animal derived reagents including FBS^18,19^. Some reports characterized ADSCs grown in culture media containing FBS or human serum, respectively^20–23^. The use of human sourced supplements is still controversial, because of its lack of availability and possibility of disease transmission between donor and patient. Serum free media (SFM), excluding animal derived components and extracts from human sources, has recently been used to produce therapeutic cells. Because of critical safety issue, use of animal-derived components in clinical cell therapy application is no longer the preferred option. Nonetheless, a comprehensive, comparative analysis of the cells cultured in FBS containing media and SFM has not yet been published. In this study, we aimed to investigate and compare the characteristics, multi-omics data, and in vivo therapeutic efficacy of ADSCs cultured in the two media and to suggest the new guide line of MSCs cultivated in SFM.

## Materials and methods

### Ethics statement

All animal experiments performed were approved by the Institutional Animal Care and Use Committee (IACUC)of Inha University and was conducted strictly under the guidelines for animal experimentation of Inha University (IACUC No. INHA 181120-600), and the ARRIVE guidelines (http://arriveguidelines.org). We have complied with all relevant ethical regulations for animal testing and research.

All ADSC experiments performed was approved by the Public Institutional Bioethics Committee designated by the Ministry of Health and Welfare (MoHW, Seoul, Korea, IRB no. P01-201903-31-008), and all methods were carried out in accordance with relevant guidelines and regulations. All patients adipose tissue samples were collected after obtaining informed consent form for human origin research on Korea National Institute for bioethics policy (KoNIBP).

### Study design

The study made use of human ADSCs collected from healthy subjects. In order to investigate the characteristics of ADSCs cultivated with SFMs, four commercial SFMs, or FBS containing media were used to analyze cells obtained from the same donor. After analysis, a comprehensive comparative analysis of ADSCs cultured with selected SFM and FBS containing media. After preparation of ADSCs from 4 healthy subjects, cellular expansion, surface markers, differentiation potency, cellular senescence, genetic stability, differential expression level of mRNA and protein, and in vivo efficacy in acute pancreatitis were compared between ADSCs cultured with SFM and ADSCs cultured with FBS containing media. The protocol for the ADSC experiments was approved by the Public Institutional Bioethics Committee designated by the Ministry of Health and Welfare (MoHW, Seoul, Korea, IRB no. P01-201903-31-008).

### Preparation of MSCs

Human adipose tissue was obtained from plastic surgeries from Dr. Jung (Dacapo Dental Hospital, Seoul, Korea). ADSCs were isolated by mechanical and enzymatical treatment of the tissue as described previously^24^. In brief, adipose tissue was minced manually into small fragments and digested with 0.075% collagenase type I (Gibco, Thermo Fisher Scientific, MA, USA) in a water bath at 37 °C. The digested tissue was centrifuged and filtered in sequential steps to separate the ADSCs from the surrounding tissue. The isolated ADSCs were expanded in low glucose DMEM supplemented with 10% FBS and 1% penicillin/streptomycin (P/S; HyClone, Cytiva, Marlborough, MA, USA), or in serum free establishment media (CellCor establishment media, Xcell Therapeutics, Seoul, Korea). After reaching 85% confluency, cells were digested with Accutase (Gibco) and seeded (3 × 10^5^ cells) in a T75 culture flask (Corning, Oneonta, NY, USA).

Human bone marrow derived MSCs (BMSCs) and human umbilical cord derived MSCs (UCSCs) was obtained from American Type Culture Collection (ATCC).

### Multipassage assay

ADSCs cultured in FBS containing media, DMEM (HyClone) supplemented with 10% FBS (HyClone), SFM (CellCor, Xcell Therapeutics). StemPro MSC SFM XenoFree (Thermo Fisher Scientific), or MesenCult-ACF Plus Culture Kit (STEMCELL Technologies) were compared. ADSCs were seeded at 4 × 10^3^ cells/cm^2^ in T25 flasks (Corning) and passaged every 4 days, except every 3 days for SFM. Flask of FBS containing media and SFM group did not need to be pre-coated. When using StemPro MSC SFM XenoFree and the MesenCult-ACF Plus Culture Kit, the flasks were pre-coated with CELLstart Substrate (Thermo Fisher Scientific) and Animal Component-Free Cell Attachment Substrate (STEMCELL Technologies), respectively. At each passage, ADSCs were detached and counted using a NucleoCounter NC-250 (ChemoMetec, Allerod, Denmark) and seeded in new T25 flasks. The population doubling time (PDT) was determined at each passage by the formula: PDT = culture period in hours / {log (number of cells T_0_) – log (number of cells T_1_)} / log(2), where T_0_ is the seeding cell number and T_1_ is the cell number at harvest. The accumulation cell number (ACN) was determined at each passage by using formula: ACN = (ACN of previous passage / T_0_) x T_1_.

### Flow cytometric surface marker expression analysis

The surface markers expression of ADSCs cultured in FBS containing media (FBS group) and ADSCs cultured in SFM (SFM group) were analyzed using a flow cytometer (Beckman Coulter, Danaher, CA, USA). Monoclonal antibodies against CD14- phycoerythrin (PE, BD Biosciences, San Diego, CA, USA), CD34-PE, CD45-PE, CD73-PE, CD90-PE, CD105-PE (BioLegend, San Diego, CA, USA), HLA-DR-PE (abcam, Cambridge, MA, UK), and Neu5Gc Fluorescien (Neu5Gc-FITC, BioLegend) were used. Fluorescence intensity of 10,000 cells per sample was recorded, and the positive expression was defined as a fluorescence greater than 99% of the corresponding unstained cell sample. Data analyses were performed using the FlowJo software (TreeStar Inc).

### Quantitative reverse transcription (qRT) PCR

Total RNA was isolated from ADSCs at the corresponding passage number by TRIzol extraction (easy-BLUE total RNA Extraction Kit, iNtRON Biotechnology, Seongnam, Korea). cDNA was synthesized by reverse transcription of 2 µg of total RNA using a cDNA synthesis kit (Promega, Madison, WI, USA), and then each gene transcript was amplified by qRT-PCR with primers. The target genes for this study were as follows: *18S rRNA* fw: GCT TAA TTT GAC TCA ACA CGG GA; *18S rRNA* rev: AGC TAT CAA TCT GTC AAT CCT GTC; *fatty acid-binding protein 4 (FABP4)* fw: AGC ACC ATA ACC TTA GAT GGG G; *FABP4* rev: CGT GGA AGT GAC GCC TTT CA; *aggrecan (ACAN)* fw: GCC TGC GCT CCA ATG ACT; *ACAN* rev: ATG GAA CAC GAT GCC TTT CAC; *osteocalcin (OCN)* fw: AAG AGA CCC AGG CGC TAC CT; *OCN* rev: AAC TCG TCA CAG TCC GGA TTG; *insulin-like growth factor-binding protein 5 (IGFBP5)* fw: AAC GAA AAG AGC TAC CGC GA; *IGFBP5* rev: CCG ACA AAC TTG GAC TGG GT; *endoglin* fw: CTT CCT GGA GTT CCC AAC G; *endoglin* rev: GGT GCC ATT TTG CTT GGA; *superoxide dismutase 2 (SOD2)* fw: GGC CTA CGT GAA CAA CCT GAA; *SOD2* rev: CTG TAA CAT CTC CCT TGG CCA; *tumor necrosis factor receptor superfamily member 11b (TNFRSF11B)* fw: GGC AAC ACA GCT CAC AAG AA; *TNFRSF11B* rev: CGC TGT TTT CAC AGA GGT CA; *pentraxin-3 (PTX3)* fw: CGA AAT AGA CAA TGG ACT CCA TCC; *PTX3* rev: CTC ATC TGC GAG TTC TCC AGC A; *colony-stimulating factor 1 (CSF1)* fw: CCA GGA ACA GTT GAA AGA TCC A; *CSF1* rev: TTA TCT CTG AAG CCA TGG TGT; *C-X-C motif chemokine ligand 12 (CXCL12)* fw: GAG CCA ACG TCA AGC ATC TG; *CXCL12* rev: CGG GTC AAT GCA CAC TTG TC; *G0G1* fw: GGC CTG ATG GAG ACT GTG TG; *G0G1* rev: CTT GCT TCT GGA GAG CCT GT.

### Multilineage differentiations

For adipogenic differentiation, ADSCs were seeded (5 × 10^5^ cells/well) in a 6-well plate (Corning) in FBS containing media or SFM and cultured for 2 days. Afterward, the medium was replaced with adipogenic induction medium (StemPro Adipogenesis Differentiation Kit, Gibco) and cultured for an additional 14 days. Cells were fixed with 4% formaldehyde for 30 min, and washed with 60% isopropanol. Cells were stained with 0.6% Oil Red O solution (Sigma-Aldrich, St Louis, MO, USA) at room temperature (RT) for 15 min. Cells were washed with 60% isopropanol and distilled water (DW).

For osteogenic differentiation, seeded cells (5 × 10^4^ cells/well, 6-well plate) were treated with osteogenic differentiation medium for 21 days, which was based on high glucose DMEM supplemented with 10% FBS, 1% GlutaMAX, 0.2 mM of ascorbic acid, 10 mM of glycerol 2-phosphate, 1% P/S, and 0.1 µM of dexamethasone. Cells were fixed as described above and washed three times with DW. Then, cells were stained with 2% Alizarin Red S solution (Sigma-Aldrich) and washed again with DW.

For chondrogenic differentiation, seeded cells (5 × 10^4^ cells/well, 6-well plate) were treated with chondrogenic differentiation medium for 14 days, which was based on DMEM containing 10% FBS, 1% P/S, 1% Insulin-transferrin-selenium-X supplement, 50 µM of ascorbic acid, 1 μM of dexamethasone, and 10 ng/mL of transforming growth factor-β (TGF-β). Cells were fixed as described above and stained with 0.5% Alcian Blue (Sigma-Aldrich).

### β-Galactosidase staining

For cellular senescence analysis, ADSCs (1 × 10^5^) were seeded in 6-well plates and cultured to 80% confluency in FBS containing media or SFM. The senescence cells Histochemical Staining Kit (Sigma-Aldrich) was used for analysis. The β-galactosidase-positive cells were captured under a bright-field microscope (TS-100, Nikon, Tokyo, Japan).

### Carboxyfluorescein succinimidyl ester (CFSE) assay

ADSCs were seeded in a 24-well plate (Corning) at a cell density of 1 × 10^4^ cells/well in FBS containing media or SFM and cultured for 1 day. CD8 + T cells were isolated from peripheral blood mononuclear cells (PBMC) using Ficoll-Paque PLUS (GE Healthcare) and the MACS CD8 + T cell isolation kit (Miltenyi Biotec). For proliferation analysis of T cells, CD8 + T cells were stained with CFSE-FITC (CellTrace CFSE Cell Proliferation Kit Protocol, Invitrogen) and treated with 20 µg/mL of phytohemagglutinin-L (PHA-L; Sigma-Aldrich) as a T cell stimulator. Stained CD8 + T cells were seeded on ADSCs at a cell density of 1 × 10^5^ cells/well and cultured for 1, 3, and 5 days. The co-cultured T cells and ADSCs in FBS containing media or SFM were analyzed by flow cytometry (Beckman Coulter). on the FITC fluorescence intensity of 10,000 cells per sample was recorded. Data analyses were performed using the FlowJo software (TreeStar Inc).

### Cytokinesis-block micronucleus assay (CBMN assay)

Cells (1 × 10^5^) were seeded on a 60 mm culture dish (Corning) and incubated at 37 °C and 5% CO_2_ overnight. After cell confluency reached about 60%, the positive control group was treated with 0.25 µg/mL of mitomycin C (mit-C; Sigma-Aldrich) dissolved in fresh medium for 24 h. On day 3, the cells were treated with 1.5 µg/mL of cytochalasin B (cyt-B; Sigma-Aldrich) and incubated overnight. After incubation, cells were harvested by centrifugation at 300xg for 5 min. The cells were gently resuspended by tapping and exposed to KCl solution (75 mM KCl:media:DW = 10: 9:1 v/v/v) within 10 min then to fresh fixative solution (methanol:glacial acetic acid = 9: 1 v/v) and allowed to stand at 4 °C for 20 min. After centrifugation at 300xg for 5 min the supernatant was removed. The fixation step was repeated three times. The cell pellet was resuspended in 40 µL of fixative solution. A drop (10–20 µl) of the cell suspension was dispensed onto a glass slide and allowed to air-dry. Slides were stained with 10% Giemsa stain (Sigma-Aldrich). Slides were prepared in octuplicate from each culture and 2,000 binucleated cells were analyzed. The binucleated cells were scored for micronuclei (MN), nucleoplasmic bridges (NPBs), and nuclear buds (NBUDs) at 15X and 100X magnifications using a digital detection scanner (Panoramic Midi, 3DHISTECH, Budapest, Hungary).

### RNA sequencing and differential gene expression analysis

Total RNA was isolated using Trizol reagent (Invitrogen). RNA quality was assessed using Agilent 2100 bioanalyzer with the RNA 6000 Nano Chip (Agilent Technologies, Amstelveen, Netherlands). RNA was quantified using ND-2000 spectrophotometer (Thermo Fisher Scientific). An RNA sequencing library was constructed from the control and test RNAs using QuantSeq 3’ mRNA-Seq Library Prep Kit (Lexogen, Inc., Austria) according to the manufacturer’s instructions. Briefly, each 500 ng of total RNA was prepared, an oligo-dT primer containing an Illumina-compatible sequence at its 5’ end was hybridized to the RNA and reverse transcription was performed. After degradation of the RNA template, second strand synthesis was initiated by a random primer containing an Illumina-compatible linker sequence at its 5’ end. The double-stranded library was purified by using magnetic beads to remove all reaction components. The library was amplified to add the complete adapter sequences required for cluster generation. The finished library was purified from PCR components. High-throughput sequencing was performed as single-end 75-bp sequencing using NextSeq 500 (Illumina, Inc., USA). QuantSeq 3’ mRNA-Seq reads were aligned using Bowtie2^25^. Bowtie2 indices were either generated from the genome assembly sequence or the representative transcript sequences for aligning to the genome and transcriptome. The alignment file was used for assembling transcripts, estimating their abundances and determining the differential expression of genes. Differentially expressed genes (DEGs) were determined based on counts from unique and multiple alignments using BEDtools^26^. The read count data were processed based on quantile normalization method using EdgeR within R (R development Core Team, 2016) using Bioconductor (Gentleman et al.,2004). Gene classification was based on searches done by DAVID (http://david.abcc.ncifcrf.gov/) and Medline databases (http://www.ncbi.nlm.nih.gov/).

### Mass spectrometry for analysis of differential protein expression

The primary cells of each donor cultured in FBS-containing media and CellCor media, respectively, were lysed in RIPA buffer with 1X protease inhibitor (cOmplete Protease Inhibitor Cocktail, Roche Diagnostics, Mannheim, Germany). The equal amounts of lysates were run on Bolt 4–12% Bis–Tris Plus Gels (Invitrogen), and stained with InstantBlue (Sigma-Aldrich). Each gel lane was cut into 8 slices before in-gel tryptic digestion following the general protocol. Briefly, protein bands were excised, destained, and washed. Proteins were reduced with 20 mM DTT and then alkylated with 55 mM iodoacetamide. After dehydration, the proteins were digested with 12.5 ng/μl of MS grade trypsin protease (Thermo Fisher Scientific) in 50 mM of ammonium bicarbonate at 37 °C overnight. Peptides were then extracted from the gel pieces serially with 10% formic acid (FA), 50% (v/v) acetonitrile (AN) in 0.1% FA and 80% AN in 0.1% FA, dried under the vacuum, and stored at − 20 °C until used. The extracted peptides were resuspended in 0.1% FA (Solvent A) and analyzed in triplicate by LC–MS/MS integrated with a Dionex 3000 UHPLC system and Q-Exactive mass spectrometer (Thermo Fisher Scientific). Peptides were loaded onto a C18 trap column (75 μm X 2 cm, 100 Å, Acclaim PepMap100, Thermo Fisher Scientific) and separated on the C18 analytical column (75 μm X 50 cm, 100 Å, PepMap RSLC, Thermo Fisher Scientific) using a 65 min linear gradient from 5–35% solvent B (0.1% FA in AN) at a flow rate of 300 nl/min. Spray voltage was set to 2.2 kV, capillary temperature to 250 °C, and the normalized collision energy to 27 eV. Q-Exactive was operated in the data-dependent acquisition mode with a MS survey scan, followed by 10 MS/MS scans of the most abundant ions. The full MS scan range was 400–1400 m*/z*, and dynamic exclusion was applied for 20 s. The collected MS/MS spectra were converted to mzXML files using the Trans-Proteomic Pipeline (version 4.8) and analyzed using the SEQUEST algorithm (version 27) in the SORCERER platform (Sage-N Research, Milpitas, CA, USA). A protein database search was performed using the UniProt human database (version 2018.08; 173,324 entries). Full tryptic specificity and up to two missed cleavage sites were allowed. Mass tolerances for precursor ions and fragment ions were set to 10 ppm and 0.5 Da, respectively. Carbamidomethyl-cysteine was designated as a fixed modification (+ 57.0215 Da) and methionine oxidation (+ 15.9949 Da) as variable modifications. All proteins with a ProteinProphet probability of ≥ 99% with a minimum of two peptides and a PeptideProphet probability of ≥ 95% were identified using Scaffold (version 4.8.9; Proteome Software, Portland, OR, USA). For relative protein quantitation which is a label-free quantitation along with spectral counts, Scaffold software allowed the MS/MS data to compare the spectral counts of identified proteins. The normalized *p*-value and signal-to-noise values for triplicate analyses of two different samples were analyzed using the Power Law Global Error Model (PLGEM) (http://www.bioconductor.org) package within R program (version 2.15). For the differentially expressed proteins (DEPs) of each donor between cells cultured in FBS containing media and CellCor media, the biological functional Data were analyzed through the use of IPA (QIAGEN Inc., https://www.qiagenbioinformatics.com/products/ingenuity-pathway-analysis).”

### Western blot

Total protein was lysed in RIPA buffer containing 1% protease inhibitor. Lysates were centrifuged at 13,000 rpm at 4 °C for 10 min and supernatants transferred to new tubes. For western blot analysis, 15 μg of protein was loaded on Bolt 4–12% Bis–Tris Plus Gels (Invitrogen). After electrophoresis, the protein was transferred to PVDF blotting membranes (Invitrogen). The membranes were blocked in 5% BSA and treated with the following primary antibodies: anti-GAPDH (1:1,000; Invitrogen), anti-filamin A (1:500; Cell Signaling Technology), anti-STAT1 (1:500; Invitrogen), anti-PTX3 (1:500; Invitrogen), anti-optineurin (1:500; Cell Signaling Technology) anti-PDZ and LIM domain 5 (PDLIM5; 1:500; Novus Biologicals), and anti-myoferlin (anti-MYOF; 1:500; Novus Biologicals), anti-small nuclear ribonucleoprotein U1 subunit 70 (anti-SNRNP70; 1:500; Invitrogen), anti-fused in sarcoma (anti-FUS; 1:500; Cell Signaling Technology), anti-Catalase (1:500; Cell Signaling Technology), anti-MCM2(1:500; Cell Signaling Technology), anti-MCM3(1:500; Cell Signaling Technology) and anti-MCM7(1:500; Cell Signaling Technology) at 4 °C for 24 h. Membranes were rinsed three times with TBS-T and treated with the secondary antibodies, anti-mouse IgG antibody (1:5000; Invitrogen) and anti-rabbit IgG antibody (1:5,000; Invitrogen), at room temperature (RT) for 1 h, then rinsed three times with TBS-T. Pico Chemiluminescent substrate reagent (Thermo Fisher Scientific) was added for enhanced detection, and the membranes were exposed to the iBright imaging system (CL1000, Thermo Fisher Scientific). Data analyses were performed using the Image J software (NIH).

### Animal experiments

All animal procedures were approved by the Institutional Animal Care and Use Committee (IACUC) of Inha University (IACUC No. INHA 181120-600). Six-week-old BALB/c mice (Charles River) were housed in pathogen-free conditions. After 12 h of fasting with free access to water, mice received intraperitoneal (IP) injections of cerulein (Sigma-Aldrich) at a dosage of 50 μg/kg body weight every 5 h to induce acute mild pancreatitis. MSCs (1 × 10^6^) were infused by tail vein injection 24 h after the last injection of cerulein. Mice were sacrificed on 3 days after MSC infusion. Blood samples were centrifuged (3,000 RPM, 4℃, 10 min), and the serum was stored at − 20 °C for analysis. The pancreas was immediately removed in a standardized fashion and divided into portions for the subsequent assays (amylase, lipase, tumor necrosis factor-α [TNF-α], interleukin-6 [IL-6]) and histologic evaluation.

Amylase activity was assessed using a commercial kit (Bioassay, Hayward, CA, USA) in which Cibacron Blue–amylose serves as a chromogenic substrate. The soluble chromogen in 0.1 ml of serum was measured spectrophotometrically at 580 nm. The absorbance was linear compared to the enzyme activity.

Plasma lipase activity was determined by the titrimetric method, also using a commercial kit (Bioassay), following the manufacturer's instructions. The principle of this method is that the degradation of triolein by lipase releases diacylglycerol, which reacts further, eventually forming hydrogen peroxide (H_2_O_2_). The H_2_O_2_ reacts with a leuco dye to form a chromophore with an absorption maximum at 412 nm. For the analysis of TNF-α and IL-6 in acute pancreatitis, we used mouse ELISA kits (R&D Systems, Minneapolis, MN, USA). The plates were coated overnight with 2 or 4 μg/ml of anti-TNF-α and IL-6 capture monoclonal antibodies (in 0.1 M Na_2_HPO_4_ pH 9 buffer) and washed with phosphate buffered saline (PBS)-Tween 20. A biotin-labeled 1 or 2 μg/ml, anti-TNF-α and anti-IL-6 detecting antibodies were used. The plates were developed using streptavidin–horseradish peroxidase (Vector, Burlingame, CA, USA) and 2, 2-azino-bis substrate (Sigma-Aldrich).

Pancreas samples were fixed in 10% buffered formaldehyde, embedded in paraffin, and sectioned. All protocols were performed following the methods of H&E staining^27^.

### Statistical analysis

All statistical analyses were performed using GraphPad Prism 5.01 (GraphPad Software, La Jolla, CA, USA). Results are presented as mean ± SEM. Differences between groups were assessed by the Mann–Whitney U test and Wilcoxon signed rank test as appropriate. Statistical significance was considered as a two-tailed at *p* < 0.05.

## Results

### Culture of ADSCs using SFM offers advantages in culturing time, multipotential capacity, genetic stability, and immunogenicity

First, we compared the proliferation, surface marker expression, and differentiation capability of ADSCs grown in different two type of media compared with FBS containing media and four SFMs. Four different SFMs and FBS containing media were used for comparative test (Supplementary Fig. 1). The cells obtained from the same donor but differed in expansion rate, surface markers expression and differentiation potential depending on the media used. Of the four SFMs used in the test, the ADSCs cultured in SFM B and SFM C were detected as partial positive for CD34 and ADSCs cultured in SFM D were detected as negative for CD90 (Supplementary Fig. 1c). Therefore, SFM, (CellCor) was chosen for the comparative analysis of FBS containing media and serum free media.

**Figure 1:**
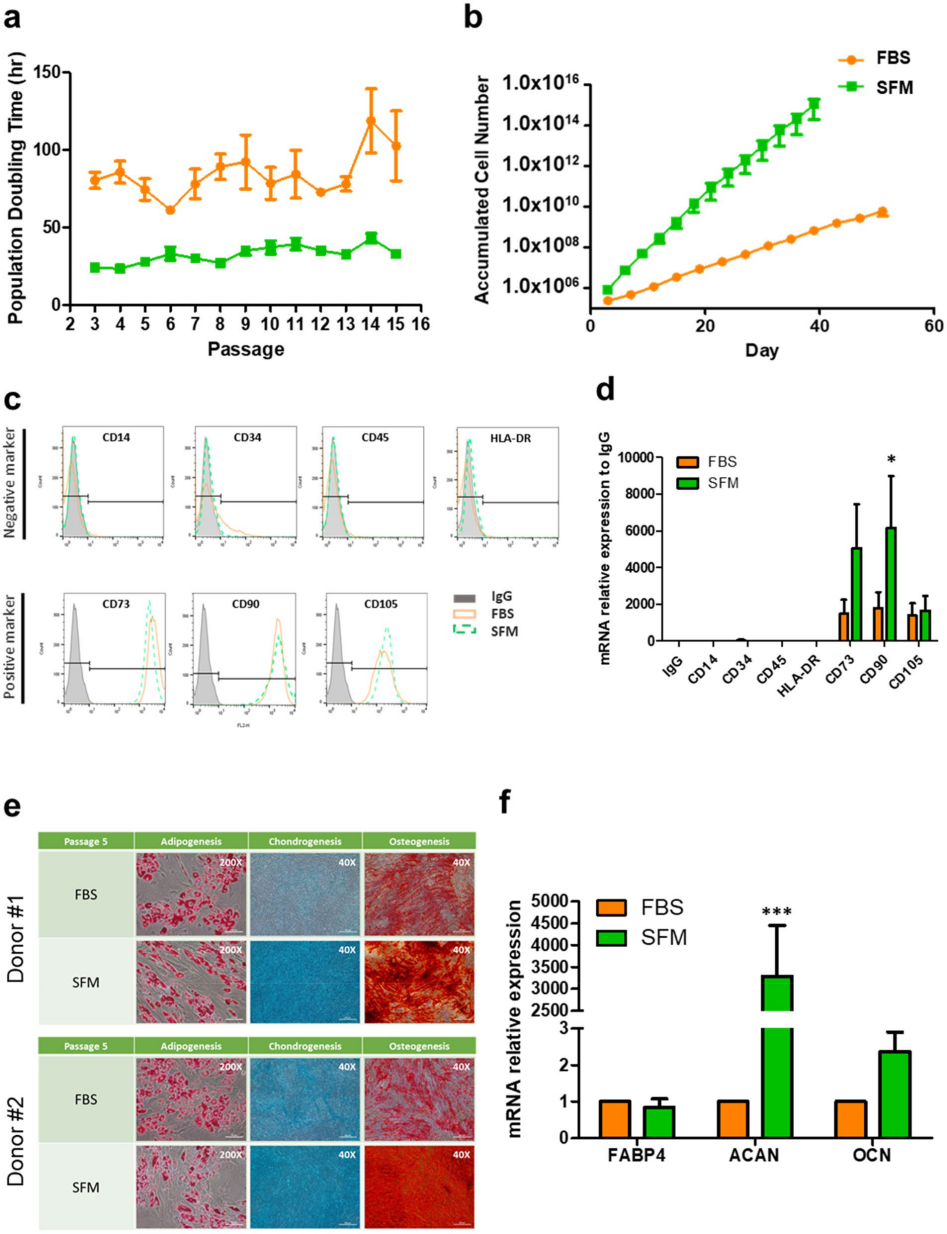
Comparison of the characteristics of ADSCs culture in SFM and FBS containing media. Calculated (**a**) population doubling time and (**b**) accumulated cell number. ADSCs cultured in serum free media (CellCor), and FBS containing media (DMEM with 10% FBS) were compared. SFM showed the lower PDT across the whole passage than FBS. At P15, SFM exhibited a higher increase in ACN than FBS. (**c**) Flow cytometric analysis of the expression surface markers of cultured ADSCs. All ADSCs showed positive expression of CD73, CD90, and CD105 and negative expression of CD14, CD34, CD45, and HLA-DR in both medium except that CD34 in FBS showed partial positive expression. A representative image from four independent experiments is shown. (**d**) Expression of surface markers by qRT-PCR analysis of total RNAs isolated from ADSCs. (**e**) Multilineage differentiation potential of ADSCs. All ADSCs were induced toward differentiation into adipocytes (verified by Oil Red O), chondrocytes (verified by Alcian Blue), and osteocytes (verified by Alizarin Red S). A representative image is shown. (**f**) qRT-PCR analysis of the mRNA expression levels of FABP4 (adipogenic marker), ACAN (chondrogenic marker), and OCN (osteogenic marker). Data represent the mean ± SEM * vs. corresponding passage FBS containing media. * *p* < 0.05, *** *p* < 0.001. *ADSC* adipose-derived stem cell, *SFM* serum free media, *FBS* fetal bovine serum, *PDT* population doubling time, *ACN* accumulated cell number, *FABP4* fatty acid-binding protein 4, *ACAN* aggrecan, *OCN* osteocalcin.

Next, ADSCs from 4 donors were cultured in SFM (CellCor) and FBS containing media (DMEM with 10% FBS), respectively, and in-depth comparative analysis was conducted. Initially, the expansion rate, expression of surface markers, and differentiation potency of ADSCs cultured in FBS containing media (ADSCs_FBS) and SFM (ADSCs_SFM) were compared. Throughout the whole passage, ADSCs_SFM showed a shorter PDT than ADSCs_FBS (Fig. 1a). At passage number (P) 15, ACN of ADSCs_SFM was about 1.09 × 10^15^, a significant increase compared to ADSCs_FBS (6.06 × 10^9^, Fig. 1b). Flow cytometry and qRT-PCR analysis were performed to detect the expression of well-known surface markers of ADSCs. The cultured ADSCs were positive for CD73, CD90, and CD105, and negative for CD14, CD34, CD45, and HLA-DR in both media, except that ADSCs_FBS showed partially positive expression of CD34, a negative marker (Fig. 1c, Supplementary Table 1). The q-RT-PCR analysis revealed significantly higher expression levels of CD90 in ADSCs_SFM than in ADSCs_FBS (Fig. 1d). The tri-lineage differentiation potency of ADSCs_FBS and ADSCs_SFM, was examined by culturing cells at P5 under conditions that support adipogenic, chondrogenic, and osteogenic differentiation. After differentiation, cells were stained as described in the methods section, and imaged under a phase-contrast microscope (Fig. 1e). The adipogenic differentiation capability was similar in both media, whereas ADSCs_SFM exhibited a superior chondrogenic and osteogenic differentiation capability when compared with ADSCs_FBS. The expression levels of each differentiation marker, including FABP4 (adipogenesis marker), ACAN (chondrogenesis marker), and OCN (osteogenesis marker) were examined by qRT-PCR analysis (Fig. 1f). The mRNA expression level of ACAN was significantly higher in ADSCs_SFM than in ADSCs_FBS. Although statistical significance was borderline (p = 0.0547), the mRNA expression level of OCN was higher in SFM than FBS/DMEM. Taken together, these results show that culture in SFM offers advantages in culturing time and multipotential capacity.

**Figure 2:**
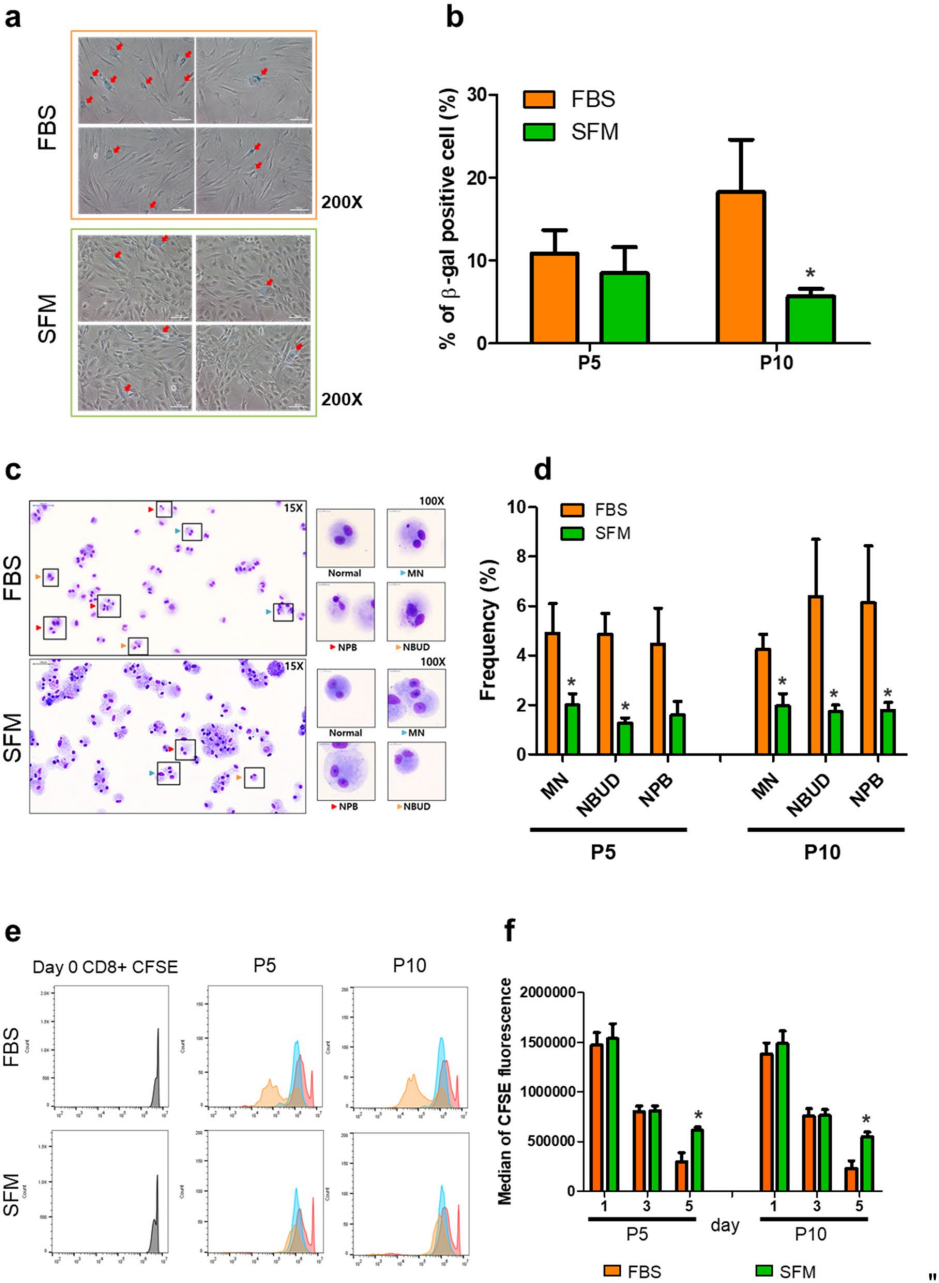
Comparison of cellular characteristics after expansion between ADSCs culture in SFM and FBS containing media. (**a**) Cellular senescence of ADSCs cultured in SFM or FBS. ADSCs at P5 and P10 were seeded and cultured for 24 h, then stained for senescence associated β-galactosidase activity. Red arrows indicate β-galactosidase positive cell; (**b**) β-galactosidase-positive cells were counted and presented as a percentage. (**c**) Genetic stability of ADSCs by cytokinesis-block micronucleus assay for MN, NBUDs, and NPBs at P5 and P10. (**d**) ADSCs cultured in FBS showed a significant increase in the frequency on MN and NBUDs formation at P5, and MN, NBUDs, and NPBs at P10 compared to ADSCs cultured in SFM. (**e**) Flow cytometric analysis of immune modulation function by co-culture of ADSCs and fluorescently labeled T cells. (**f**) Fluorescence intensity per cell after co-culture reflected the functional ability of ADSCs to suppress T cell proliferation. T cell proliferation was inhibited more by ADSCs cultured in SFM than ADSCs cultured in FBS. Data are represented as the mean ± SEM. * vs. corresponding passage FBS containing media. * *p* < 0.05. *ADSC* adipose derived stem cell, *SFM* serum free media, *FBS* fetal bovine serum, *MN* micronuclei, *NBUD* nuclear bud, *NPB* nucleoplasmic bridge.

ADSCs are usually expanded by in vitro culture before application. At this stage, ADCSs could be exposed to various stresses, including physical detachment, temperature change, and cell damage induced by protease, leading to cellular senescence. Measurements were taken of the most common marker of senescence the lysosomal enzyme, senescence-associated β-galactosidase (SA-β-gal) at pH6^28^. Senescence levels between ADSCs_SFM and ADSCs_FBS were compared at P5 and P10 (Fig. 2a,b). About 11% of ADSCs_FBS (10.8 ± 2.9%) and 9% of ADSCs_SFM group (8.5 ± 3.1%) were shown to be positive for SA-β-gal at P5. Interestingly, the SA-β-gal positive cell population at P10 was 18.3 ± 6.3% and 5.7 ± 0.9% in ADSCs_FBS and ADSCs_SFM, respectively. This result indicates that culture in SFM has an advantage in reducing cellular senescence of ADSCs compared to FBS containing media.

The rapid growth rate of cells means that the division rate of the nucleus is relatively fast, which can induce genetic instability. Hence, the genetic stability of cells was examined and compared by the CBMN assay^29,30^. MN, NBUDs, and NPBs were evaluated (Fig. 2c,d). At P5 and P10, ADCSs_FBS showed a significant increase in the formation frequency of MN, NBUDs, and NPB compared to ADSCs_SFM, which showed a significant decrease in all three variables, except that at P5, the NBP formation of ADSCs_SFM was not significantly different from ADSCs_FBS. In particular, ADSCs_ FBS exhibited an increase in NBUDs an NPBs at P10 compared to P5. This result is evidence that culture in SFM is more genetically stable compared to FBS containing media.

MSCs display unique immunomodulatory properties by direct or indirect manner^31^. Usually, MSCs secrete anti-inflammatory cytokine to suppress activated immune cell. However, animal factors derived from FBS can be internalized into cells and processed, then presented as antigen on cell surface, resulting in the proliferation of immune cells. Because MSCs are known to be partial positive for MHC-I and negative for MHC-II, we analyzed inhibition of CD8^+^ T cell proliferation by ADCSs_FBS and ADSCs_SFM, respectively. This regulatory function can be assayed in vitro when fluorescently labeled T cells were treated with PHA-L, and co-cultured with ADSCs. Flow cytometric analysis of the fluorescence intensity per cell after co-culture, serves as an indirect way to quantify the functional ability of ADSCs to suppress T cell proliferation (Fig. 2e,f). These results show that T cell proliferation was inhibited more in SFM than in FBS containing media at P5 and P10. In addition, ADSCs cultured in FBS containing media are more immunogenic than ADSCs cultured in SFM, which limits repeated treatment in clinical trial.

### A comparative multi-omics analysis of ADSCs_FBS and ADSCs_SFM

**Figure 3:**
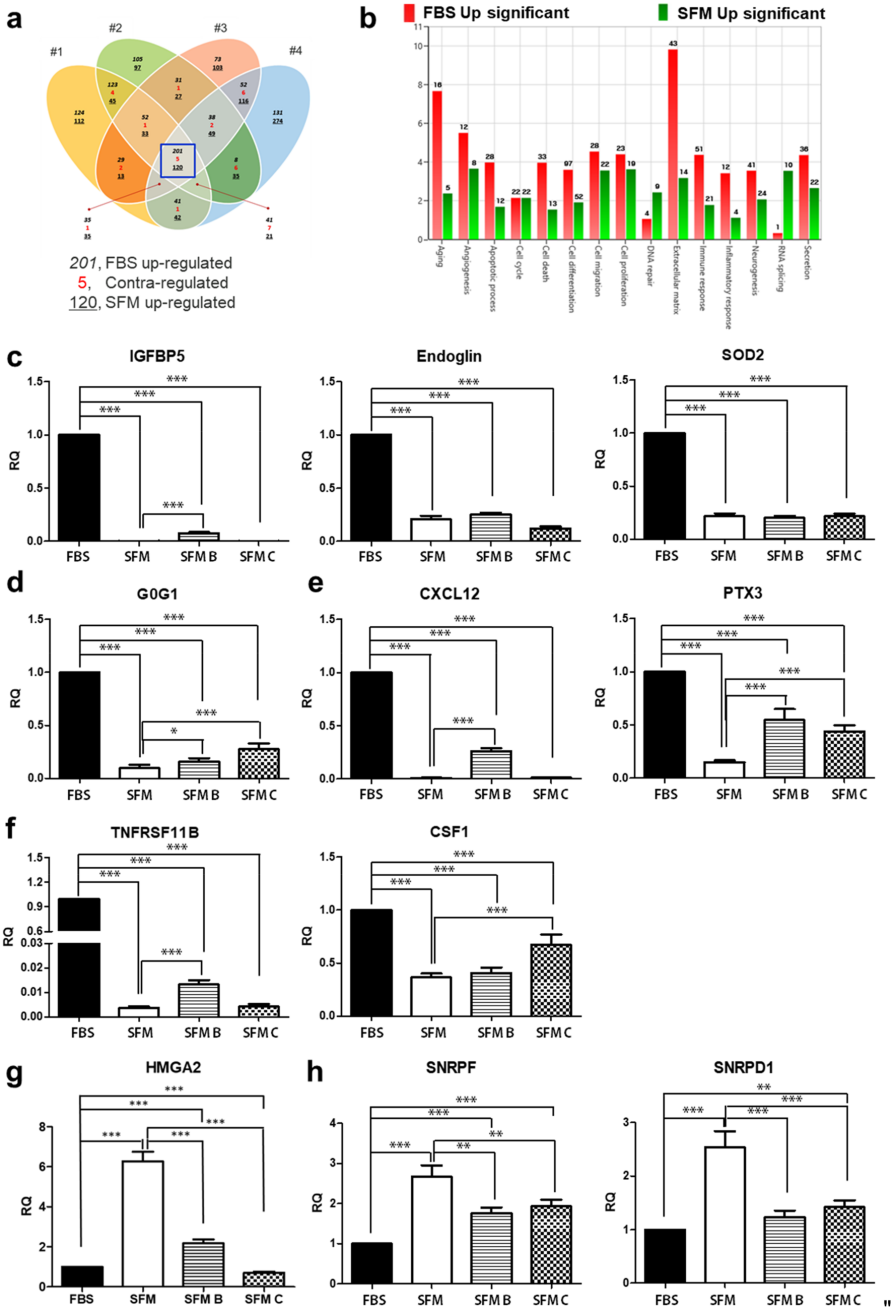
Comparison of mRNA expression profile. The levels of mRNA expression of ADSCs cultured in SFM and FBS containing media were compared by RNA sequencing. (**a**) mRNA expression levels in ADSCs from four donors grown in the different media, showed up-regulation of 120 genes in ADSCs cultured in SFM and 201 genes in ADSCs cultured in FBS. (**b**) Gene ontology terms enriched by differentially expressed genes after cell expansion in the two different media. ADSCs cultured in FBS showed up-regulation of genes involved in aging, apoptosis, cell death, extracellular matrix, immune response, and inflammatory response. Gene expression profile validated by qRT-PCR analysis after cell expansion in the FBS containing media and three different SFMs included (**c**), aging (**d**), apoptosis (**e**), immune response (**f**), inflammatory response (**g**), DNA repair (**h**), and RNA splicing. Data are represented as the mean ± SEM. ** *p* < 0.01, *** *p* < 0.001. *ADSC* adipose-derived stem cell, *SFM* serum free media, *FBS* fetal bovine serum, *IGFBP5* insulin-like growth factor binding protein 5, *SOD2* superoxide dismutase 2, *CXCL12* C-X-C motif chemokine ligand 12, *PTX3* pentraxin-3, *TNFRSF11B* tumor necrosis factor receptor superfamily member 11B, *CSF1* colony-stimulating factor 1, *HMGA2* high mobility group A2 protein, *SNRPF* small nuclear ribonucleoprotein polypeptide F, *SNRPD1* small nuclear ribonucleoprotein D1, *SFM, SFM B, and SFM C* are CellCor, StemPro, and MesenCult, respectively.

To determine why ADSCs_FBS and ADSCs_SFM have different growth rates, differentiation, cellular senescence, genetic stability and immunogenicity, we performed a multi-omics analysis, including DEG and DEP. To determine the media dependent changes of mRNA expression, mRNA sequencing analyses were carried out. Global change in mRNA expression profiles that were commonly observed in the four donors were identified. Of the 321 DEGs obtained (fold change > 2), 201 of genes were up-regulated in ADSCs_FBS and 120 of genes were up-regulated in ADSCs_SFM. Functional annotation of these DEGs, was performed using DAVID, a common tool for GO enrichment analysis. Up-regulated functional gene categories in ADSCs_FBS included aging, apoptosis, cell death, extracellular matrix, immune response, and inflammatory response. The genes related to RNA splicing and DNA repair were up-regulated in ADSCs_SFM (Fig. 3a,b). RNA splicing is a prerequisite for stable RNA formation^32^. A representative gene was selected from each gene category, and the differential gene expression between ADSCs cultivated in FBS containing media and three SFMs (SFM, SFM B, and SFM C) was measured. The expression of IGFBP5, endoglin, and SOD2 in the gene categories affected in ‘aging’ gene category were significantly lower all SFMs (SFM, SFM B, and SFM C) cultivated ADSCs than in ADSCs_FBS (Fig. 3c). G0G1 in the ‘apoptosis’ gene category, CXCL12 and PTX3 in the ‘immune response’ gene category, TNFRSF11B and CSF1 in the ‘inflammatory response’ gene category were significantly down-regulated in ADSCs cultured in all SFMs than FBS containing media (Fig. 3d–f). Conversely, ADSCs cultured in all SFM showed a significant up-regulation of high mobility group A2 protein (HMGA2) in the ‘DNA repair’ gene category and small nuclear ribonucleoprotein polypeptide F (SNRPF) in the ‘RNA splicing gene’ category than ADSCs cultured in FBS containing media (Fig. 3g,h). Small nuclear ribonucleoprotein D1 (SNRPD1) in the ‘RNA splicing’ gene category was significantly up-regulated in ADSCs cultured in SFM and SFM C than FBS containing media, but not in ADSCs cultured in SFM B (Fig. 3h).

**Figure 4:**
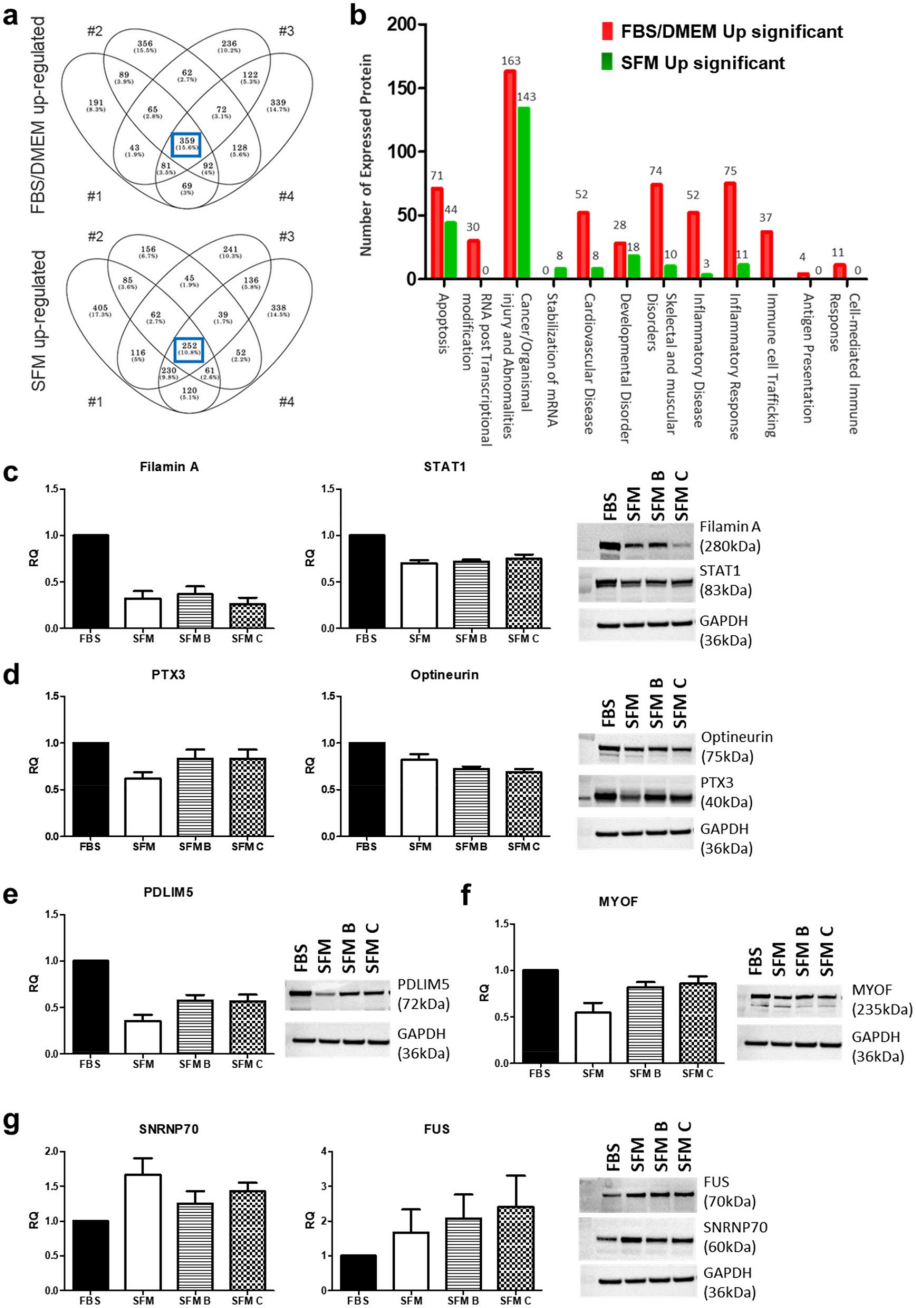
Comparison of protein expression profile. Protein identification and abundance were detected by LC–MS/MS. (**a**) Protein expression in ADSCs from four donors grown in the two different media, showed up-regulation of 252 proteins in ADSCs cultured in SFM and 356 proteins in ADSCs cultured in FBS. (**b**) Up-regulated proteins in group FBS were involved in apoptosis, cardiovascular disease, skeletal and muscular disorders, inflammatory response, immune cell trafficking, and cell-mediated immune response. Functional classification analysis validated by western blot after cell expansion in the FBS containing media and three different SFMs included (**c**) apoptosis, (**d**) inflammatory response, (**e**) cardiovascular disease, (**f**) skeletal and muscular disorders, (**g**) mRNA stabilization. Data are represented as the mean ± SEM. *ADSC* adipose-derived stem cell, *SFM* serum free media, *FBS* fetal bovine serum, *STAT1* signal transducer and activator of transcription 1, *PDLIM5* PDZ and LIM domain 5, *MYOF* Myoferlin, *SNRNP70* small nuclear ribonucleoprotein U1 subunit 70, *SFM, SFM B, and SFM C* are CellCor, StemPro, and MesenCult, respectively.

DEPs were identified by LC–MS/MS analysis. About 11% and 16% of the proteins were up-regulated in ADSCs_SFM and ADSCs_FBS, respectively. Up-regulated functional proteins categories in ADSCs_FBS included apoptosis, cardiovascular disease, skeletal and muscular disorders, inflammatory response, immune cell trafficking, and cell-mediated immune response. ADSCs_SFM showed up-regulation of functional proteins categories involved in stabilization of mRNA (Fig. 4a,b). Representative proteins from the previously reported references were selected from each protein category, and the levels of DEPs in ADSCs cultured in FBS containing media and three SFMs were compared by western blot analysis. The expression of filamin A and STAT1 in ‘apoptosis’ protein category and PTX3 and optineurin in ‘inflammatory response’ protein category tend to be lower in ADSCs cultured in all SFM than in FBS containing media (Fig. 4c,d). The expression of PDLIM5 in the ‘cardiovascular disease’ protein category and MYOF in the ‘skeletal and muscular disorders’ protein category were down-regulated in ADSCs cultured in all SFM than in FBS containing media (Fig. 4e,f). The expression levels of SNRNP70 and FUS in the ‘stabilization of mRNA’ protein category was up-regulated in ADSCs cultured in all SFM than in FBS containing media (Fig. 4g).

### Comparison of therapeutic efficacy of ADSCs_SFM and ADSCs_FBS in acute pancreatitis mouse model

**Figure 5:**
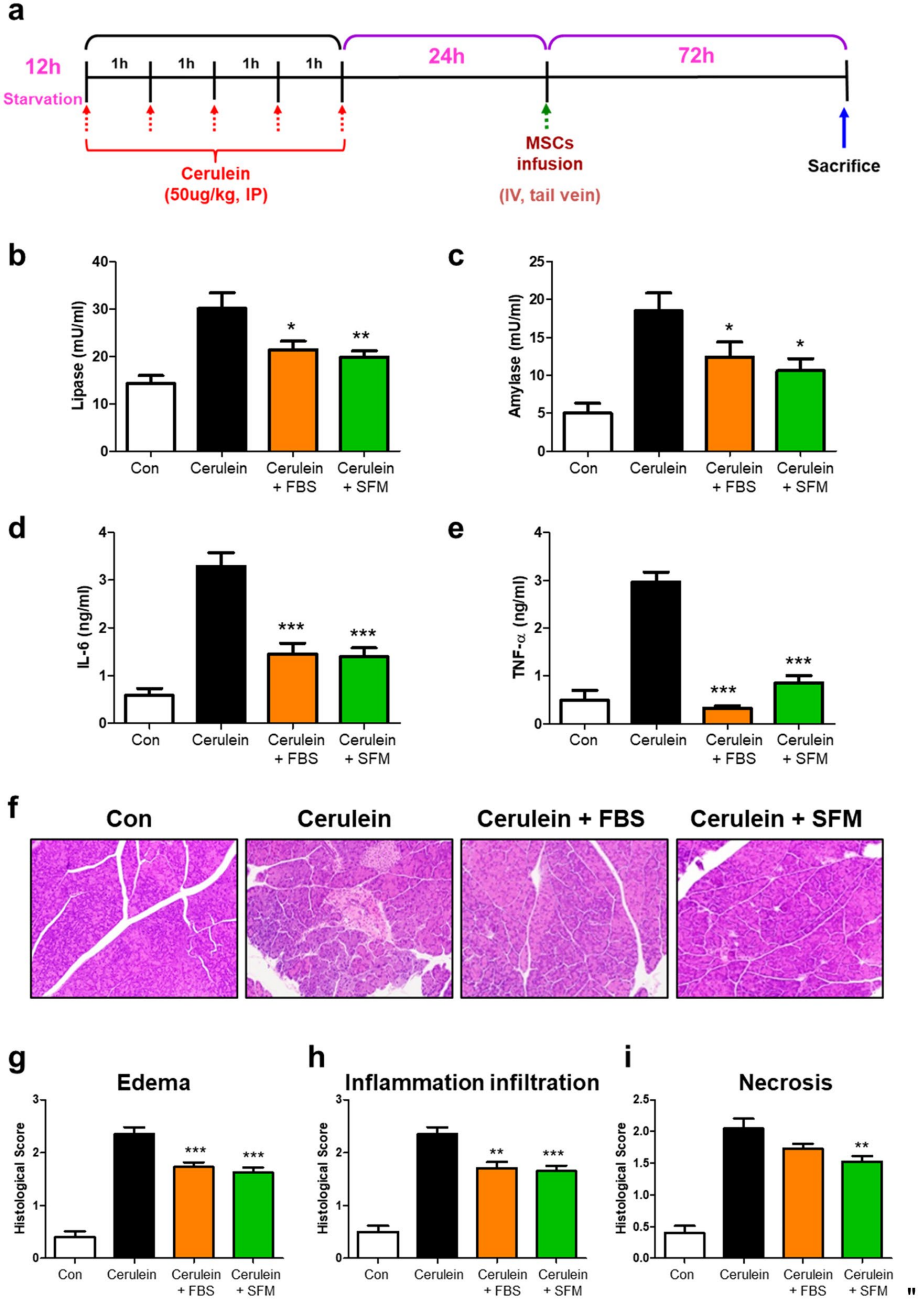
Effects of ADSCs in cerulein induced pancreatitis in mice. (**a**) Acute pancreatitis was induced by cerulean administered five times. After 24 h, ADSCs were infused to investigate their therapeutic efficacy. (**b**,**c**) Activities of lipase and amylase were significantly decreased after ADSC infusion. (**d**,**e**) Inflammatory cytokines (IL-6 and TNF-α) were significantly decreased after ADSC infusion, as detected by ELISA. Edema formation, inflammation infiltration, and necrosis were evaluated by histologic analysis. (**f**) Representative image of mouse pancreas after H&E staining. (**g–i**) Histologic changes in pancreas isolated on day 4 after last cerulein injection. Data are represented as the mean ± SEM. * vs. cerulein-only injection group * *p* < 0.05, ** *p* < 0.01, *** *p* < 0.001. *ADSC* adipose-derived stem cell, *MSC* mesenchymal stem cell, *Con* control, *FBS* fetal bovine serum, *SFM* serum free media.

Pancreatitis has obvious indicators, such as pancreatic edema and high levels of amylase and lipase. The levels of serum lipase and amylase were quantified to evaluate the severity of pancreatitis (Fig. 5a). Both enzymes decreased by about 29% and 33% after infusion of ADSCs_FBS and by about 34% and 43% after infusion of ADSCs_SFM (Fig. 5b,c). The effects of the infusion of ADSCs on the production of pro-inflammatory cytokines (IL-6 and TNF-α) linked to acute pancreatitis were examined. ADSCs_SFM as well as ADSCs_FBS significantly suppressed production of IL-6 and TNF-α, suggesting that ADSCs efficiently decreased inflammatory response in vivo (Fig. 5d,e). Histopathological analyses showed that cerulein induced significant mass edema and inflammation with necrosis compared to normal control (Fig. 5f). The infusion of ADSCs_SFM as well as ADSCs_FBS significantly decrease edema formation, inflammatory cell infiltration, and necrosis (Fig. 5g–i). Human ADSCs are xenogenic cells in mice, differences in therapeutic efficacy could not be discerned in this animal model, but whether or not the inclusion of FBS-derived antigen in culture medium altered the therapeutic effect of ADSCs was not the objective of this experiment. Taken together, this animal experiment sought to confirm that ADSCs_SFM have a therapeutic effect at least comparable with ADSCs_FBS.

## Discussion

One of big hurdles in stem cell culture is to ensure high levels of growth without loss of cell phenotype and characteristics. The characterization studies presented here demonstrate the difference in cell productivity between FBS containing media and SFM and proposed new standards. A comparative analysis of cell growth, cellular characteristics, multi-omics, and in vivo efficacy of ADSCs cultured in the two different media was undertaken. From our results, the benefits of stem cell expansion in SFM instead of FBS-containing media are that the cells can be grown at a faster rate while maintaining lower cellular senescence and higher genetic stability, resulting in relatively large number of cells, and stable cells could be obtained. Unlike other adult stem cells, MSCs can be easily isolated from many tissues, besides adipose, including bone marrow, and umbilical cord^33,34^. The cell growth, culture characteristics, and cellular senescence of bone marrow derived mesenchymal stem cells (BMSCs) and umbilical cord derived mesenchymal stem cells (UCSCs) were also assessed (Supplementary Fig. 2 and Supplementary Fig. 3). BMSCs and UCSC cultured in SFM (BMSCs_SFM, UCSCs_SFM) showed a shorter and more stable PDT than their counter parts culture in FBS containing media (BMSCs_FBS, UCSCs_FBS). BMSCs_ SFM showed relatively weak adipogenesis potential but stronger potential in chondrogenesis and osteogenesis. UCSCs_SFM showed relatively strong osteogenesis. The cellular senescence level was shown to be reduced in BMSCs _SFM and un UCSCs_SFM at P7 and P15. Cultivation time and cellular senescence were relatively superior for the BMSCs and UCSCs cultured in SFM, a trend similar to that of the ADSCs.

Some studies have indicated that murine and human MSCs exhibit reduced differentiation potential upon prolonged in vitro culture^35–38^. Previous studies have shown that senescent MSCs can cause functional changes in stem cell therapy^39^. The literatures suggest that many factors secreted from senescent MSC are able to systemically induce inflammatory response, reduce the immune modulation activity of administered hMSC and promote the proliferation or migration of cancer cells. Similarly, our results also revealed that ADSCs_FBS which showed relatively higher cellular senescence levels, had reduced immune suppression (Fig. 2), suggesting that when MSCs cultured in animal factor containing media are clinically administered, its therapeutic efficacy might be limited by offset effect due to immunogenicity even though in vivo therapeutic efficacy could not be differentiated due to the limitations of the experimental animal model by xenogenic environment. Therefore, it is thus important to expand MSCs while maintaining a low cellular senescence level.

In cells cultured with SFM, high cell proliferation rates were identified during the same culture period (Fig. 1). During rapid cell division, if the nucleus is not completely divided, this can lead to loss of genetic material in the nucleus. However, in this study, it was confirmed that the CBMN analysis and nucleus were stably divided at a rapid growth rate. In the CBMN assay, MN results from chromosomal breakage and/or loss caused by errors in either DNA repair or chromosomal segregation The NPBs indicate the occurrence of rearrangements in which chromatids or chromosomes are pulled to opposite poles during anaphase. NBUDs are alterations that indicate amplified DNA removed from the cell nucleus and are known as a marker of gene amplification^30^. NBUDs can also form when an NPB between two nuclei breaks and the remnants shrink back toward the nuclei. Furthermore, NBUDs can occur temporarily after the NPB breaks^40^. Consistent with these definitions, our results also demonstrated that the ADSCs_FBS which had a higher frequency of MN formation, showed lower gene expression levels associated with DNA repair than ADSCs_SFM (Fig. 2c,d). Previous studies have shown that NBUDs and MN influence on MSC growth^7^. Notably, it was previously found that the number of NBUDs was inversely proportional to the rate of cell proliferation. In addition, they suggested that it needs to confirm the incidence ratio of NBUDs and MN to examine genetic stability of cell therapy products as part of quality control for MSCs to be applied as an effective cell therapy product. In agreement with the previous findings, our data showed that the cell growth of ADSCs_FBS, which had a higher frequency of MN and NBUD formation, was slower than that of ADSCs_SFM. Not only that, in the analysis of chromosomes through Karyotyping, fewer number abnormal chromosomes were observed in the ADSCs_SFM group compared to the ADSCs_FBS group (data not shown). Cell therapeutics using MSCs are being continuously developed and marketed. In addition, the area of gene cell therapeutics is expected to rapidly grow in the near future. Since gene cell therapeutics should be critically considered for the genetic stability, and for this reason, SFM would give advantages in production of cell-based therapeutics.

Cell culture media that include FBS have been used to develop cell therapeutics. However, due to various problems, including genetic stability, there are ongoing attempts to replace FBS^41^. Human serum and HPL is being used as the alternatives to FBS. It has been demonstrated that human serum and HPL could support human MSC expansion in vitro^23,42^. HPL contains a variety of chemokines that regulate the migration, and proliferation of MSCs. It has been used as a culture medium supplement for clinical applications of MSCs^43,44^. However, there are limited resources of HPL, and the batch variation of HPL preparation affects the phenotype and function of MSCs. HPLs are acquired and pooled from several donors, causing variation of production batches. HPL free SFM can avoid such problems because they enable consistent product manufacturing and standardization^45–47^. We tested the inclusion of animal serum-derived substances and human serum-derived substances in ADSCs cultivated in FBS containing media and three different SFMs (Supplementary Fig. 4). Neu5Gc was used as the marker of the animal serum-derived substance^14^, and BDKRB1 as the marker of the human serum-derived substance^48^. All tested SFM did not contain animal serum-derived substances, but BDKRB1, human serum-derived substance marker, showed significantly high expression in SFM B (Supplementary Fig. 4b). ADSCs cultured in SFM B showed a higher mRNA expression of CXCL12, PTX3, and TNFRSF11B compared to other SFMs (SFM and SFM C; Fig. 3e,f). This finding suggests the possibility of induction of immunogenicity or an inflammatory reaction due to human serum-derived substances.

In addition, the serum-derived components used in cell culture contain extracellular vesicles (EVs), including serum-derived exosomes, and the EVs contained in serum can affect clinical results as well as experimental results due to lot or batch variation^49^. EVs derived from FBS or human serum, for example, induce the cancer cell growth or contaminate exosomes derived from stem cells^50,51^. In concurrence with these adverse effects, our results show that the expression of proteins involved in the functional categories of ‘cardiovascular disease’ and ‘skeletal and muscle orders’ was high in ADSCs_FBS containing media. Similar problems may be observed in cultures containing cell-derived serum and platelets. Therefore, careful consideration is required when choosing a SFM because of the potential presence of human-derived materials (e.g., HPL).

## Conclusion

We have shown that the gene and protein expression of cultured cells may vary depending on the medium used. In addition, MSCs cultured in FBS containing media showed high immunogenicity, genetic instability and senescence compared to those cultured in SFM. The results support the importance of using SFM for cell-based therapeutics and also suggest new criteria for various characteristics of MSCs cultured in SFM.

## Supplementary Information

Supplementary Information 1.

Supplementary Information 2.

Supplementary Information 3.

Supplementary Information 4.

Supplementary Information 5.

Supplementary Information 6.

## Data Availability

All mRNA expression sequencing data are available in Gene Expression Omnibus (GEO) at accession number GSE200086 (https://www.ncbi.nlm.nih.gov/geo/query/acc.cgi?acc=GSE200086). All other data are available from the authors upon reasonable request.
