# Supplementary material for: Comparative analysis of mesenchymal stem cells cultivated in serum free media
Source: Sci Rep. 2022 May 21;12:8620. doi: 10.1038/s41598-022-12467-z (PMC9124186; doi:10.1038/s41598-022-12467-z)

Fig. 4 (c)

* N/A
− Not applicable

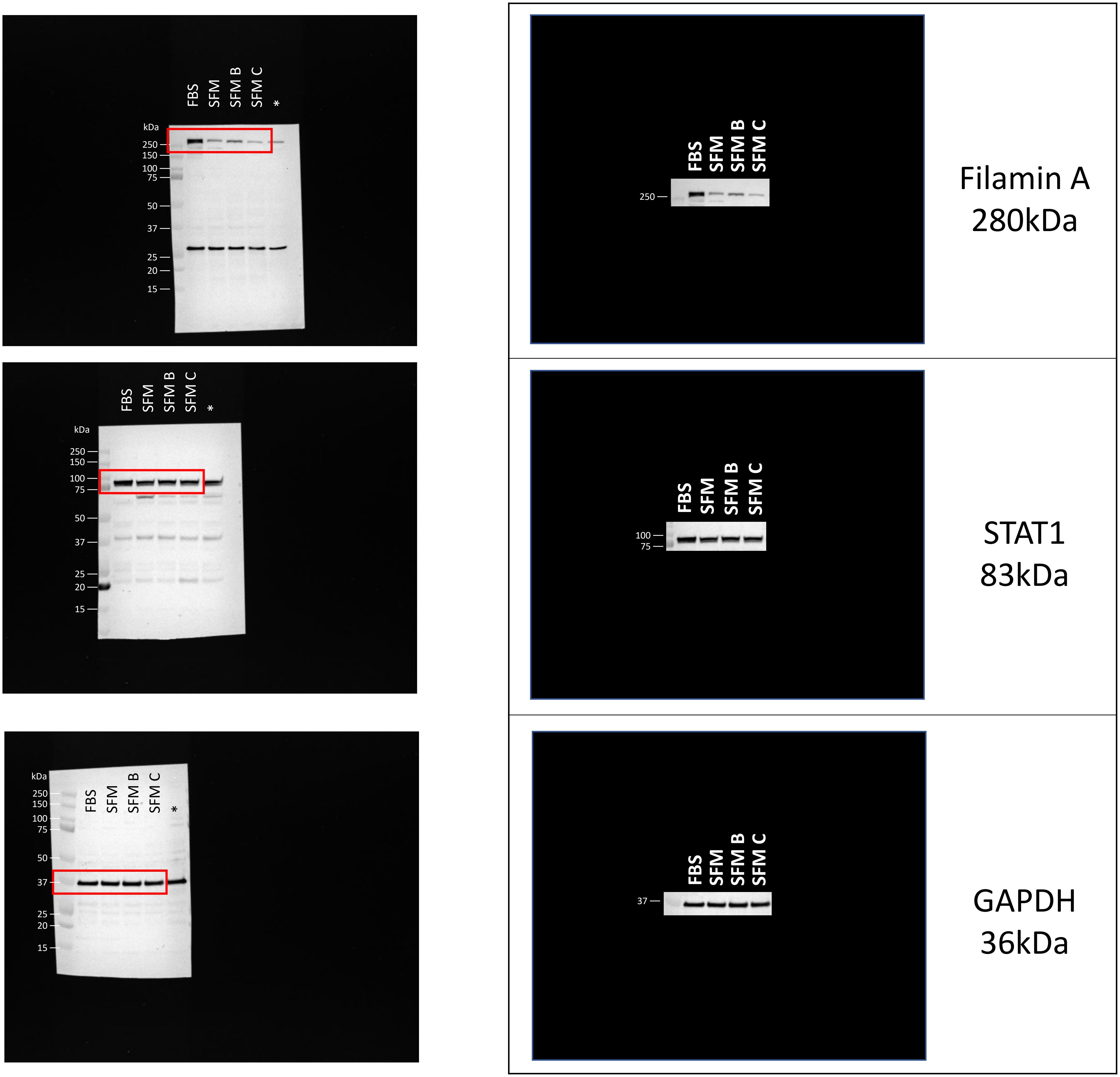

Fig. 4 (d)

*** N/A**
– Not applicable

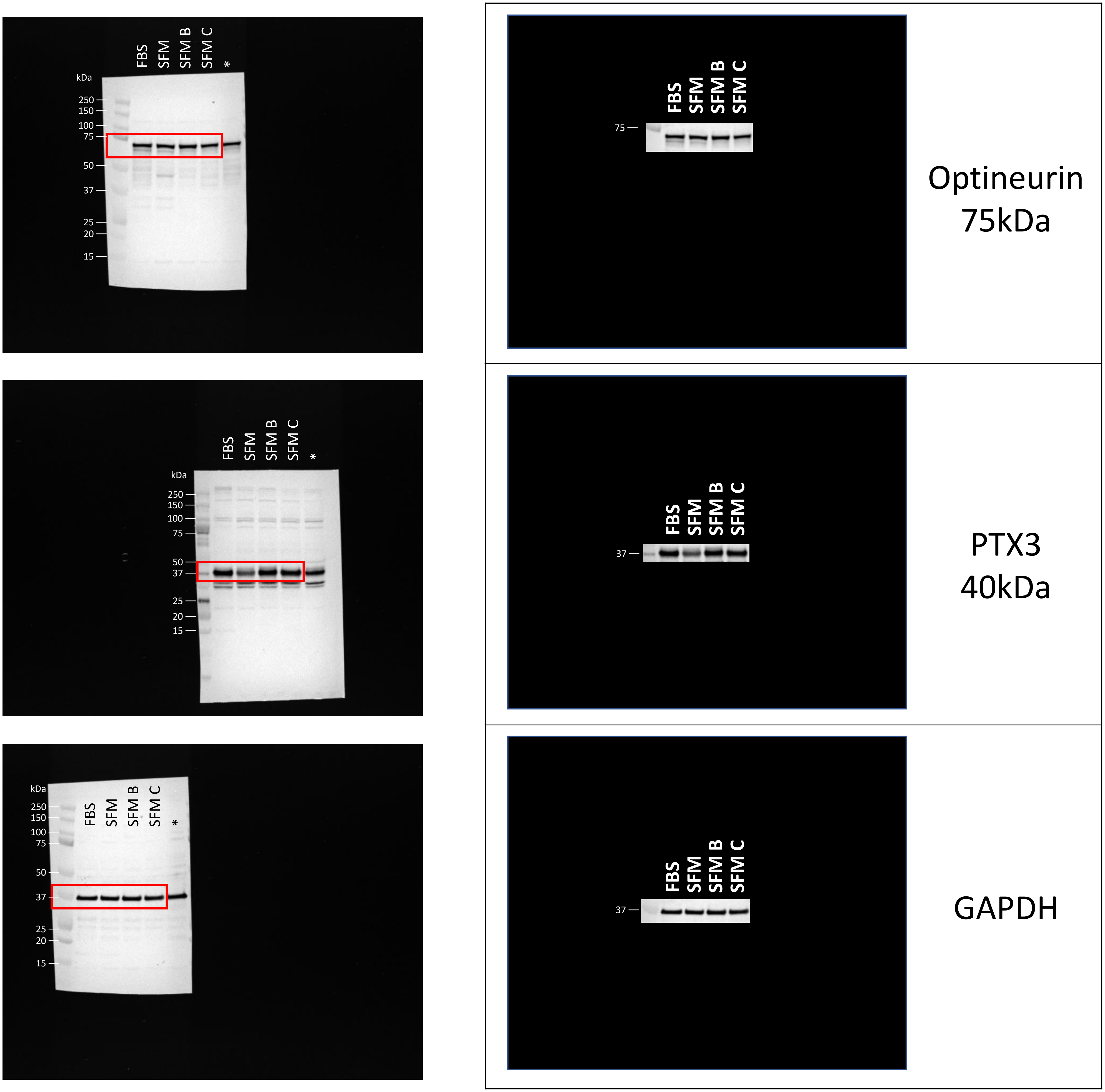

Fig. 4 (e)

*** N/A**
– Not applicable

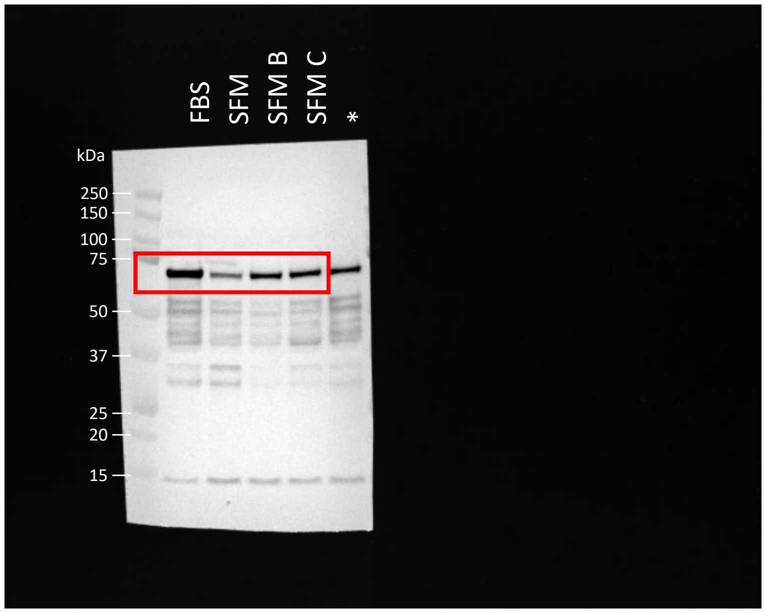

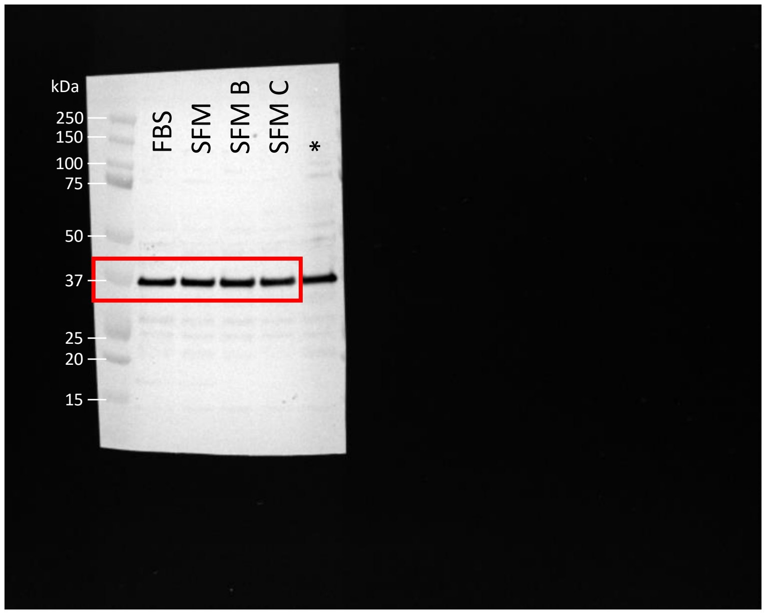

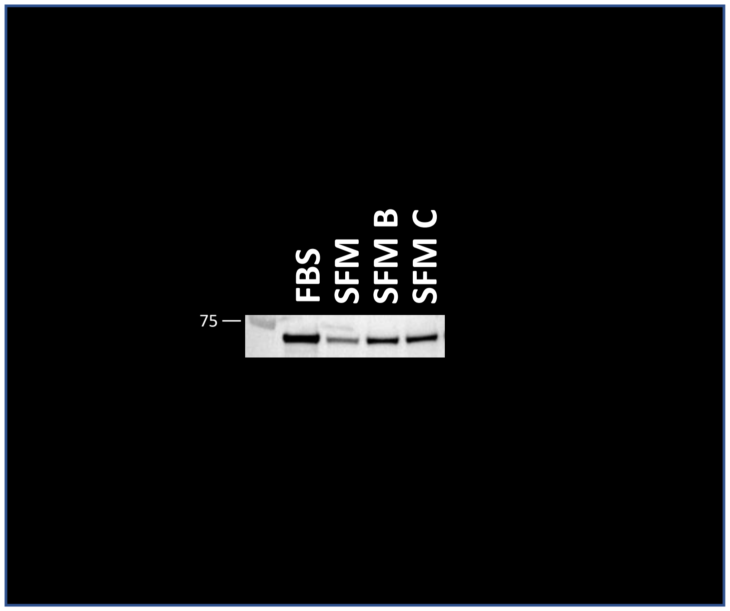

PDLIM5
72kDa

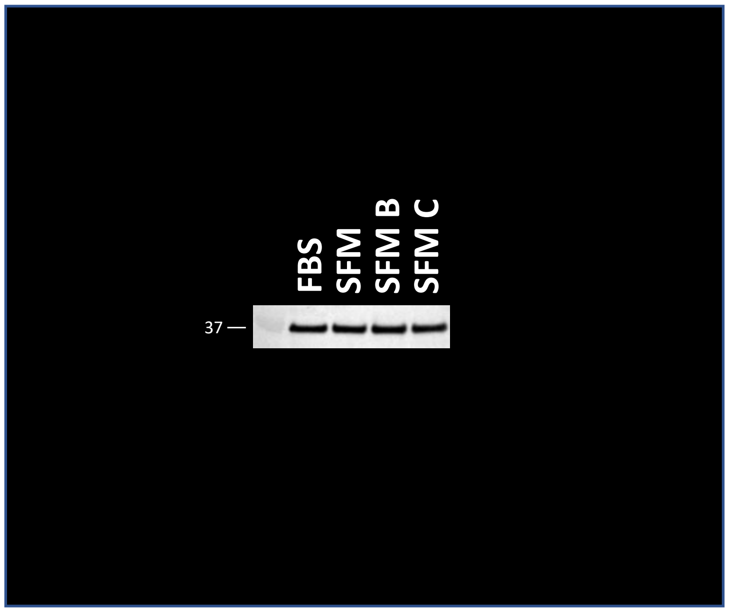

GAPDH
36kDa

Fig. 4 (f)

* N/A
– Not applicable

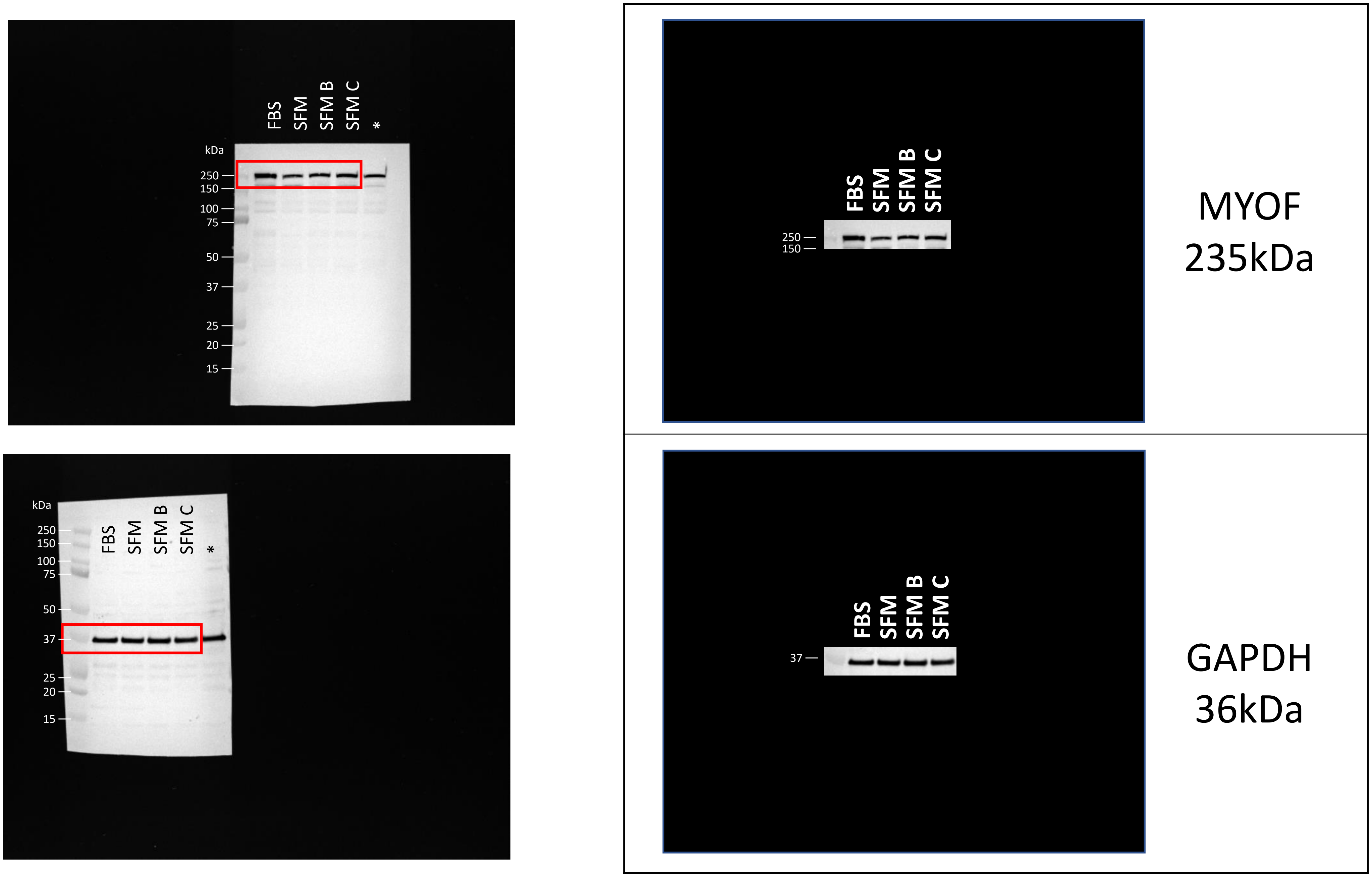

Fig. 4 (g)

*** N/A**
– Not applicable

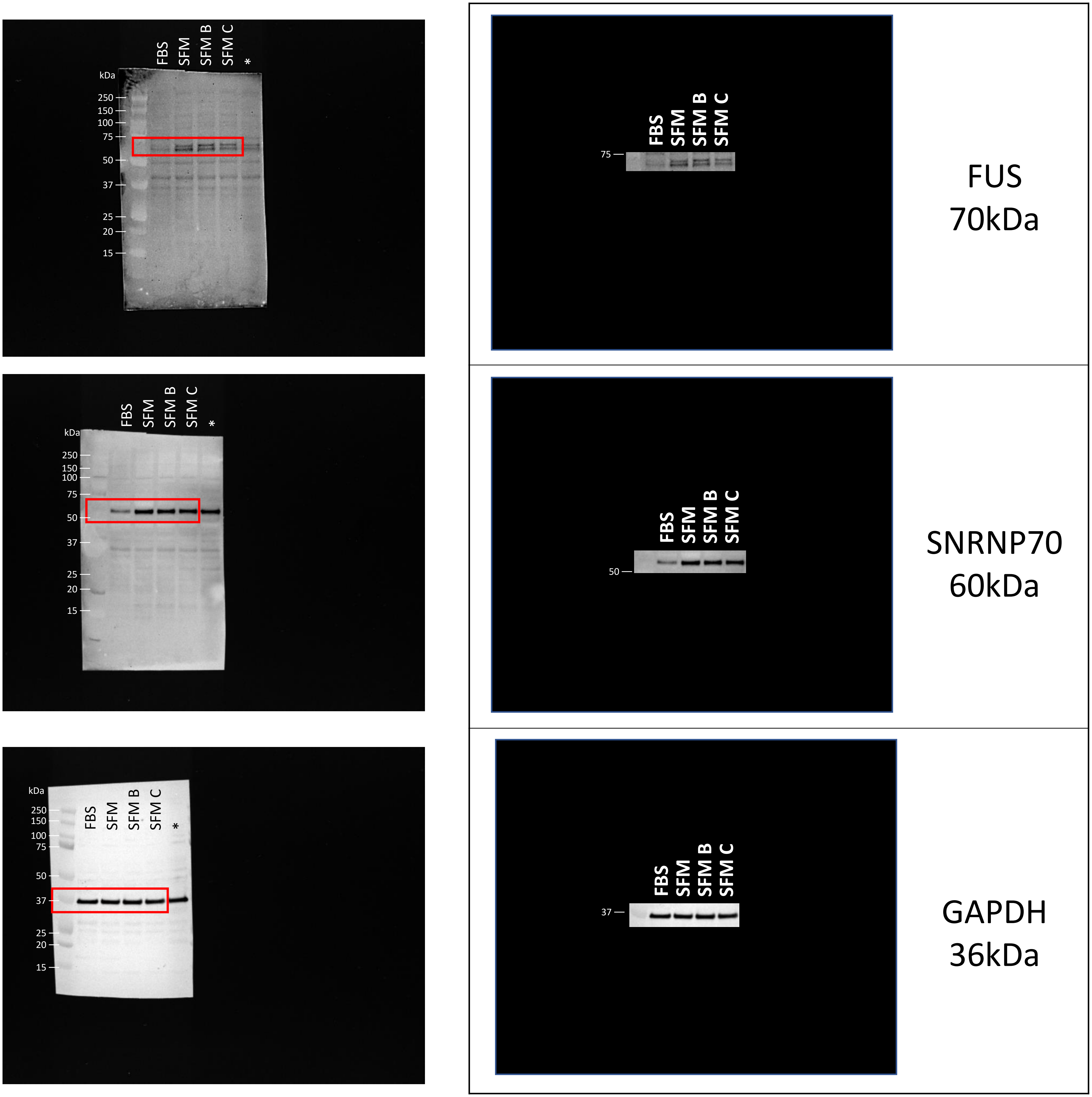

Supplement: Supplementary file 1 — Supplementary Information 1. [file 41598_2022_12467_MOESM1_ESM.pdf]
