# Supplementary material for: Comparative analysis of mesenchymal stem cells cultivated in serum free media
Source: Sci Rep. 2022 May 21;12:8620. doi: 10.1038/s41598-022-12467-z (PMC9124186; doi:10.1038/s41598-022-12467-z)
Supplement: Supplementary file 2 — Supplementary Information 2. [file 41598_2022_12467_MOESM2_ESM.docx]

**
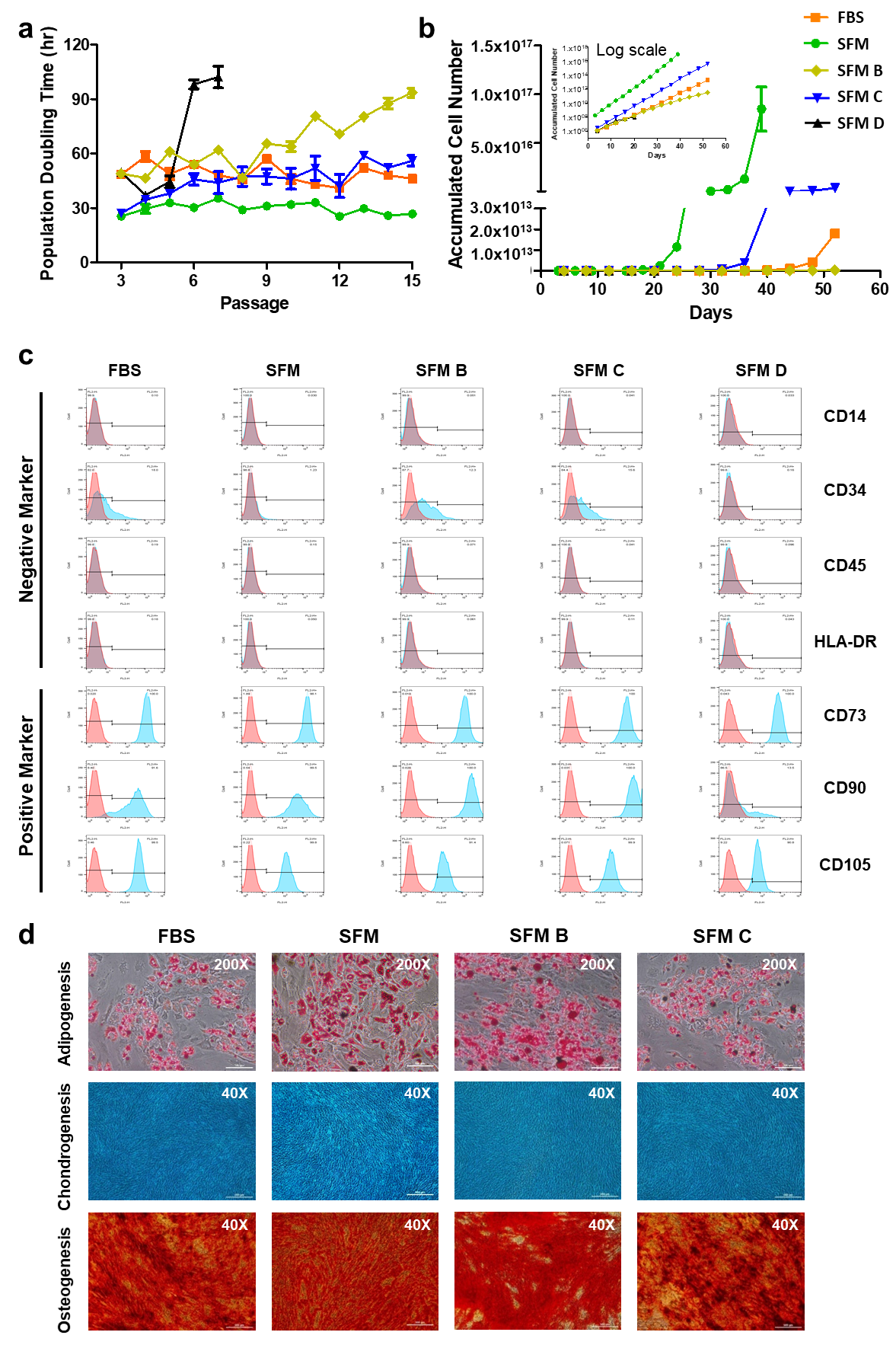
**

**Supplementary Fig. 1 | Comparison of ADSC characteristics after cultivation in FBS containing media and four SFM.** Calculated **a**, PDT and **b**, ACN. ADSCs cultured in SFM showed the lowest PDT and highest ACN across the entire passage among the test groups. ACN of ADSCs cultured in SFM exhibited the highest increase compared to that in FBS. ADSCs culture with SFM D had very little expansion and could no longer be cultivated after P7. **c**, Flow cytometric analysis of expression of surface markers of cultured ADSCs. Cells were obtained from the same donor but differed in expression of surface markers depending on the media used. **d**, Multilineage differentiation potential of ADSCs were tested. ADSC, adipose-derived stem cell; FBS, fetal bovine serum; SFM, serum-free media; PDT, population doubling time; ACN, accumulated cell number. SFM, SFM B, SFM C, and SFM D are CellCor, StemPro, MesenCult, and CSTi, respectively.
