# Supplementary material for: Comparative analysis of mesenchymal stem cells cultivated in serum free media
Source: Sci Rep. 2022 May 21;12:8620. doi: 10.1038/s41598-022-12467-z (PMC9124186; doi:10.1038/s41598-022-12467-z)
Supplement: Supplementary file 3 — Supplementary Information 3. [file 41598_2022_12467_MOESM3_ESM.docx]

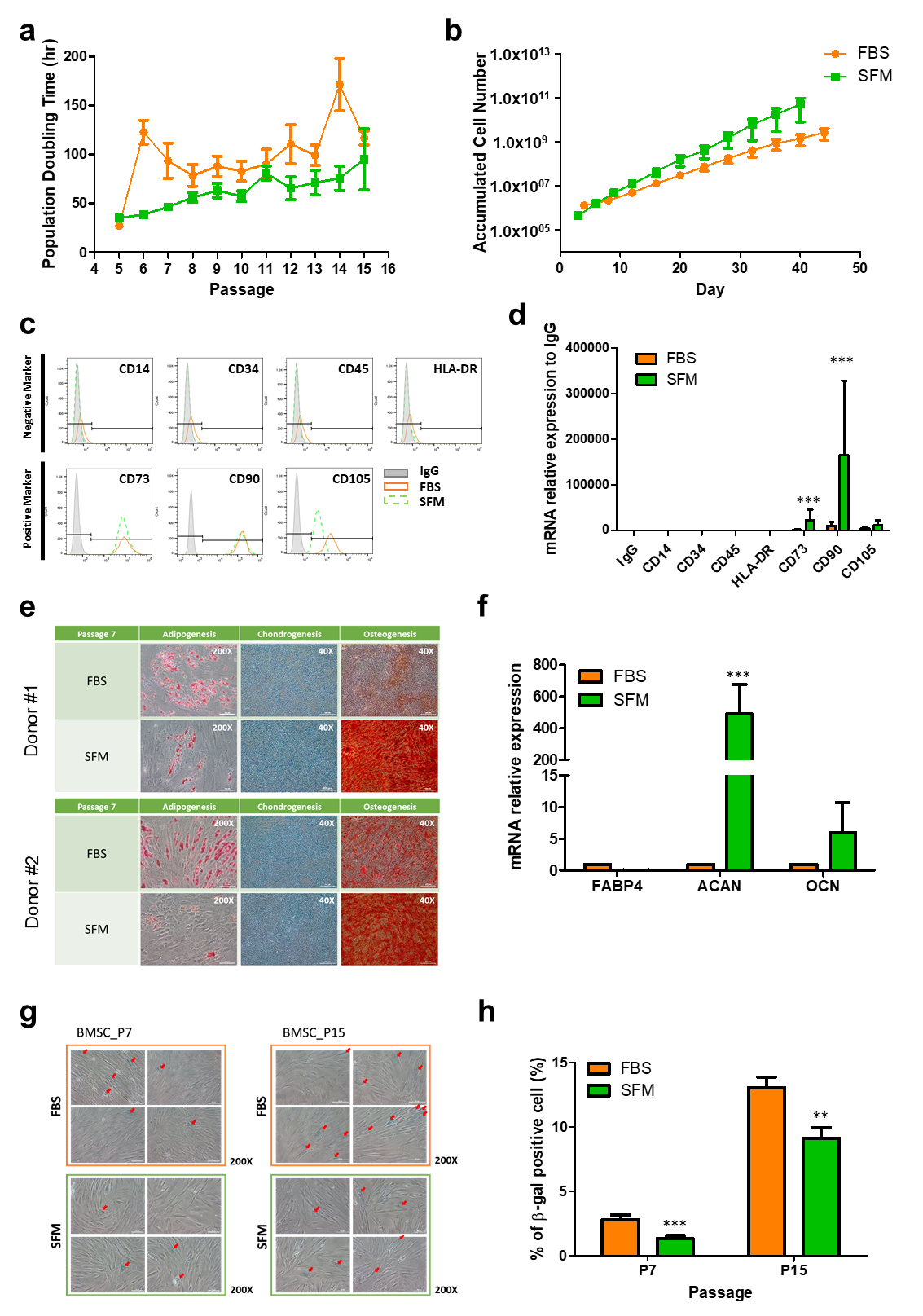


**Supplementary Fig. 2 | Comparison of BMSC characteristics after cultivation in SFM and FBS containing media.** Calculated **a**, PDT and **b**, ACN. BMSCs from three donors cultured in SFM showed the lower PDT across the entire passage than that in FBS. At P15, BMSCs cultured in SFM exhibited a higher increase in ACN compared to that in FBS. **c**, Flow cytometric analysis of expression of surface markers of cultured BMSCs. BMSCs were positive for CD73, CD90, and CD105 and negative for CD14, CD34, CD45, and HLA-DR in BMSCs cultured with both SFM and FBS. A representative image from ndependent experiments is shown. **d**, Expression of surface markers by qRT-PCR analysis of isolated total RNAs. The expression of CD73 and CD90 was significantly up-regulated in BMSCs cultivated in SFM. Multilineage differentiation potential of BMSCs cultured in FBS and SFM were examined. **e,** All BMSCs were induced toward differentiation into adipocytes (verified by Oil Red O), chondrocytes (verified by Alcian Blue), and osteocytes (verified by Alizarin Red S). A representative image is shown. **f**, qRT-PCR analysis of relative mRNA expression levels of FABP4, ACAN, and OCN. ACAN expression was significantly up-regulated in BMSCs cultured in SFM. **g**, Cellular senescence. BMSCs at P7 and P15 were seeded and cultured for 24h, then stained for senescence-associated β-galactosidase activity. Red arrows indicate β-galactosidase positive cell; **h**, β-galactosidase positive cells were counted and presented as a percentage. Data represent the mean ± SEM * vs. corresponding passage FBS containing media. * *p* < 0.05, *** *p* <0.001. BMSC, bone marrow-derived stem cell; SFM, serum-free media; FBS, fetal bovine serum, PDT, population doubling time; ACN, accumulated cell number; BMSCs, bone marrow derived stem cells; FABP4, fatty acid binding protein 4; ACAN, aggrecan; OCN, osteocalcin.
