# Supplementary material for: Comparative analysis of mesenchymal stem cells cultivated in serum free media
Source: Sci Rep. 2022 May 21;12:8620. doi: 10.1038/s41598-022-12467-z (PMC9124186; doi:10.1038/s41598-022-12467-z)
Supplement: Supplementary file 4 — Supplementary Information 4. [file 41598_2022_12467_MOESM4_ESM.docx]

**
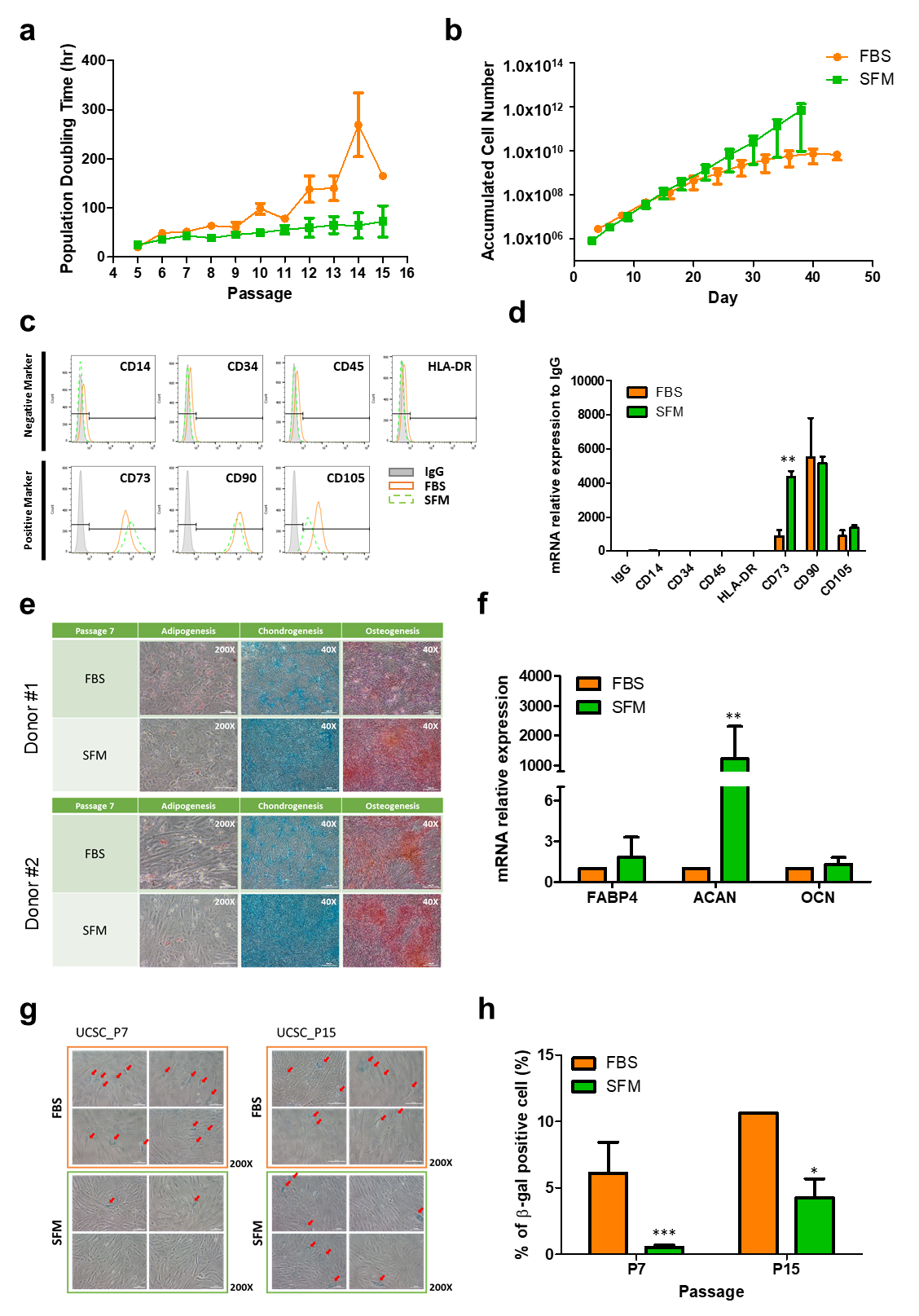
**

**Supplementary Fig. 3 | Comparison of UCSC characteristics after cultivation in SFM and FBS containing media.** Calculated **a**, PDT and **b**, ACN. UCSCs from three donors cultured with SFM showed the lower PDT across the entire passage compare to that in FBS. At P15, UCSCs cultured in SFM exhibited a higher ACN increase than that in FBS. **c**, Flow cytometric analysis of expression of surface markers of cultured UCSCs. UCSCs were positive for CD73, CD90, and CD105 and negative for CD14, CD34, CD45 in both media. A representative image from experiments is shown. **d**, Expression of surface markers by qRT-PCR analysis of isolated total RNAs. CD73 expression was significantly up-regulated in UCSCs cultured in SFM. Multilineage differentiation potential of UCSCs. **e**, All UCSCs were induced toward differentiation into adipocytes (verified by Oil Red O), chondrocytes (verified by Alcian Blue), and osteocytes (verified by Alizarin Red S). A representative image is shown. **f**, qRT-PCR analysis of relative mRNA expression levels of FABP4, ACAN, and OCN. ACAN expression was significantly up-regulated in UCSCs cultured in SFM. **g**, Cellular senescence of UCSCs. UCSCs at P7 and P15 were seeded and cultured for 24 h, then stained for senescence-associated β-galactosidase activity. Red arrows indicate β-galactosidase-positive cells; **h**, β-galactosidase-positive cells were counted and presented as a percentage. Data represent the mean ± SEM * vs. corresponding passage FBS containing media. * *p* < 0.05, *** *p* < 0.001. UCSC, umbilical cord derived stem cell; SFM, serum free media; FBS, fetal bovine serum; PDT, population doubling time; ACN, accumulated cell number; FABP4, fatty acid binding protein 4; ACAN, aggrecan; OCN, osteocalcin.
