# Supplementary material for: Comparative analysis of mesenchymal stem cells cultivated in serum free media
Source: Sci Rep. 2022 May 21;12:8620. doi: 10.1038/s41598-022-12467-z (PMC9124186; doi:10.1038/s41598-022-12467-z)
Supplement: Supplementary file 5 — Supplementary Information 5. [file 41598_2022_12467_MOESM5_ESM.docx]

**
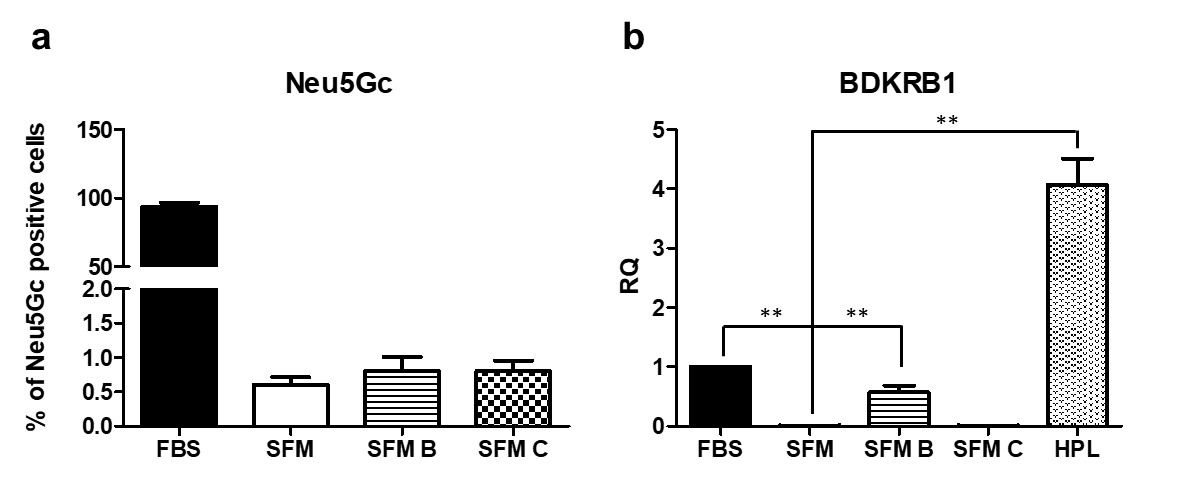
**

**Supplementary Fig. 4 | Expression of non-human serum related marker and human serum related marker in cultured ADSCs. a,** Flow cytometric analysis of expression of Neu5Gc, marker for animal serum derived substance of cultured ADSCs in FBS containing media and three different SFMs. b, qRT-PCR analysis of relative mRNA expression levels of BDKRB1, marker for human serum derived substance of cultured ADSCs in FBS containing media and three different SFMs. Cells were obtained from the same donor but differed in expression of surface markers depending on the media used. Data represent the mean ± SEM. ** *p* < 0.01. ADSC, adipose-derived stem cell; Neu5Gc, N-glycolylneuraminic acid; BDKRB1, mammalian bradykinin receptor subtype B1; SFM, serum-free media; FBS, fetal bovine serum. SFM, SFM B, and SFM C are CellCor, StemPro, and MesenCult, respectively.
