# Supplementary material for: Comparative analysis of mesenchymal stem cells cultivated in serum free media
Source: Sci Rep. 2022 May 21;12:8620. doi: 10.1038/s41598-022-12467-z (PMC9124186; doi:10.1038/s41598-022-12467-z)
Supplement: Supplementary file 6 — Supplementary Information 6. [file 41598_2022_12467_MOESM6_ESM.docx]

**Supplementary Table 1 |** Expression of surface markers by flow cytometric analysis

| **Donor** | **Media** | **CD14** | **CD34** | **CD45** | **HLA-DR** | **CD73** | **CD90** | **CD105** |
| --- | --- | --- | --- | --- | --- | --- | --- | --- |
| ADSC01 | FBS | 1.20 | 17.30 | 0.80 | 0.70 | 99.58 | 99.79 | 99.96 |
|  | SFM | 3.50 | 1.40 | 0.40 | 0.60 | 99.99 | 99.97 | 99.99 |
| ADSC02 | FBS | 0.90 | 48.50 | 0.70 | 0.70 | 99.94 | 98.40 | 99.90 |
|  | SFM | 0.10 | 0.50 | 0.10 | 0.00 | 99.98 | 94.94 | 99.68 |
| ADSC03 | FBS | 1.20 | 14.60 | 1.10 | 1.20 | 99.93 | 99.81 | 99.73 |
|  | SFM | 0.20 | 2.90 | 0.40 | 0.80 | 100.00 | 99.90 | 99.95 |
| ADSC04 | FBS | 0.00 | 36.70 | 0.10 | 25.90 | 99.89 | 99.77 | 99.71 |
|  | SFM | 0.00 | 0.00 | 0.70 | 0.00 | 99.72 | 99.93 | 99.82 |

FBS, fetal bovine serum; SFM, serum free media
